# Highly Informative Single-Copy Nuclear Microsatellite DNA Markers Developed Using an AFLP-SSR Approach in Black Spruce (*Picea mariana*) and Red Spruce (*P. rubens*)

**DOI:** 10.1371/journal.pone.0103789

**Published:** 2014-08-15

**Authors:** Yong-Zhong Shi, Natascha Forneris, Om P. Rajora

**Affiliations:** 1 Forest Genetics and Biotechnology Group, Department of Biology, Dalhousie University, Halifax, Nova Scotia, Canada; 2 Faculty of Forestry and Environmental Management, University of New Brunswick, Fredericton, New Brunswick, Canada; University of Massachusetts, United States of America

## Abstract

**Background:**

Microsatellites or simple sequence repeats (SSRs) are highly informative molecular markers for various biological studies in plants. In spruce (*Picea*) and other conifers, the development of single-copy polymorphic genomic microsatellite markers is quite difficult, owing primarily to the large genome size and predominance of repetitive DNA sequences throughout the genome. We have developed highly informative single-locus genomic microsatellite markers in black spruce (*Picea mariana*) and red spruce (*Picea rubens*) using a simple but efficient method based on a combination of AFLP and microsatellite technologies.

**Principal Findings:**

A microsatellite-enriched library was constructed from genomic AFLP DNA fragments of black spruce. Sequencing of the 108 putative SSR-containing clones provided 94 unique sequences with microsatellites. Twenty-two of the designed 34 primer pairs yielded scorable amplicons, with single-locus patterns. Fourteen of these microsatellite markers were characterized in 30 black spruce and 30 red spruce individuals drawn from many populations. The number of alleles at a polymorphic locus ranged from 2 to 18, with a mean of 9.3 in black spruce, and from 3 to 15, with a mean of 6.2 alleles in red spruce. The polymorphic information content or expected heterozygosity ranged from 0.340 to 0.909 (mean = 0.67) in black spruce and from 0.161 to 0.851 (mean = 0.62) in red spruce. Ten SSR markers showing inter-parental polymorphism inherited in a single-locus Mendelian mode, with two cases of distorted segregation. Primer pairs for almost all polymorphic SSR loci resolved microsatellites of comparable size in *Picea glauca*, *P. engelmannii*, *P. sitchensis*, and *P. abies*.

**Significance:**

The AFLP-based microsatellite-enriched library appears to be a rapid, cost-effective approach for isolating and developing single-locus informative genomic microsatellite markers in black spruce. The markers developed should be useful in black spruce, red spruce and other *Picea* species for various genetics, genomics, breeding, forensics, conservation studies and applications.

## Introduction

Microsatellites or simple sequence repeats (SSR) are highly informative genetic markers. Due to their co-dominant inheritance, high polymorphism, reproducibility and transferability across related species, microsatellites have been widely used for various genetics, genomics, breeding, genetic resource conservation, and forensics studies and applications, e.g., [1]–[8]. Although, recently with the use of next-generation sequencing (NGS) technologies, genome-wide single nucleotide polymorphism (SNP) and other markers have become available, microsatellite markers are still highly suitable for population and conservation genetics studies and forensics applications, especially for determining neutral population genetic processes and dynamics. In fact, microsatellites were demonstrated to provide more precise information on population genetic structure than SNPs in four different taxa [9]. Also, highly polymorphic co-dominant markers, such as microsatellites, are needed to align genetic maps.

Various methods and strategies have been used to isolate microsatellite-containing sequences in plants and animals, which are primarily based on the development of microsatellite-enriched and non-enriched genomic libraries [10]. Also, availability of a large number of expressed sequence tag (EST) sequences in the database made it possible to identify microsatellite sequences and develop cDNA-based microsatellite markers in agricultural and horticultural plants, e.g., [11]–[13] and conifer trees [14], [15]. The advantages associated with EST-derived microsatellite markers include high probability of such markers being single-locus types and their ability to detect polymorphism directly in expressed genes. However, EST-derived microsatellite markers show lower polymorphism than genomic microsatellites [7]. This is possibly due to lower mutation rates, conservation and slow evolution of gene sequences and selection constraints on them. Therefore, information obtained from EST-derived microsatellites is likely to be different from that obtained from faster-evolving genomic microsatellites. Also, the expressed portion of the genome is quite small, especially in plants with large genome size; thus, cDNA-based microsatellite markers may have limited and non-random representation of the genome. Hence, it is important to develop genomic microsatellites. More recently, massive parallel sequencing of the whole transcriptomes and genomes can reveal a large number of sequences with microsatellite repeats.

Due primarily to huge genome size (2–4×10^10^ base pairs) and highly repetitive genomes of conifers [16], [17], development of highly informative single-locus genomic microsatellite markers remains a challenge in these long-lived plants. Although a large number of microsatellite-containing sequences can now be identified by whole genome sequencing in conifers [18], [19] using NGS platforms, the challenge still remains to isolate and develop genomic microsatellite markers that show simple single-locus DNA variant patterns. Genomic microsatellite markers have been developed for a number of spruce (*Picea*) and other conifer species using non-enriched and microsatellite-enriched [2], [20]–[23] and BAC/YAC genomic libraries [24], [25]. However, the number of genomic microsatellites showing single-copy single-locus patterns remains small because a large proportion of genomic microsatellites have shown complex multilocus patterns with a frequent presence of null alleles. The difficulties and challenges have been mainly attributed to the large genome size and the presence of high proportion of redundant repetitive DNA sequences throughout the genomes in conifers [16], [17]. In order to minimize the problems, attempts were made to isolate microsatellite markers from unique DNA sequences, using two approaches: low copy enrichment method [26] and undermethylated (UM) DNA method following methylation restriction enzyme (*Mcr*BC) [27]. Of the above two approaches, the low-copy enrichment method was found to be more successful. However, both of these methods are still time consuming and expensive and yielded only a small number of highly informative single-locus microsatellite markers. Development of informative single-locus microsatellite markers was however more successful from cDNA or EST sequences in conifers [13]–[15].

Here we report a simple and efficient approach for the development of highly informative single-locus genomic microsatellite markers in two widely distributed and economically and ecologically important conifers, black spruce (*Picea mariana*) and red spruce (*Picea rubens*). Microsatellite sequences were identified from microsatellite-enriched AFLP DNA fragments in black spruce. We have determined informativeness of these markers in black spruce and red spruce, their Mendelian single-locus inheritance and linkage in progeny of two controlled crosses of black spruce, and their cross-species transferability to four other spruce species.

## Materials and Methods

### Plant Material and DNA Extraction

One of the parents (accession # 59) of a three-generation outbred mapping pedigree of black spruce was used for the construction of microsatellite-enriched library. Thirty individuals each of black spruce and red spruce drawn from 12 populations were used to determine the informativeness of the microsatellite markers. Eight individuals each of white spruce (*P. glauca*), Sitka spruce (*P. sitchensis*), Engelmann spruce (*P. engelmannii*), and Norway spruce (*P. abies*) were used for determining the cross-species transferability of the microsatellite markers, with the exception of one marker RPMSA07 for which five individuals of each species were used. The black spruce samples were previously obtained from nine populations located at three sites in Manitoba, Canada [28] as follows: Pine Falls – natural mature (1), post-harvest natural young regeneration (1), and plantation (4); Bissett – natural mature (3), post-harvest natural young regeneration (2), and plantation (3); and Snow Lake – natural mature (3), post-harvest natural young regeneration (6), and plantation (7). The red spruce samples were from three provenances included in a range-wide provenance test established at the Acadia Forest Experiment Station, Fredericton, New Brunswick: Great Smoky Mountain National Park, Tennessee-North Carolina boundary (6), Monongahela National Forest, Pocahontas County, West Virginia (12), and Logan State Forest, Pennsylvania (12). The Norway spruce samples were obtained from the Nova Scotia Department of Natural Resources, Sitka spruce samples from the British Columbia Ministry of Natural Resources, and white spruce and Engelmann spruce samples were from Alberta, Canada. The parents and 110, and 30 progeny of two *F*_2_ controlled crosses of black spruce 32×40, and 46×14, respectively, were used to examine the inheritance and linkage of microsatellite markers. The details on controlled crossing experiments and pedigrees are provided in Kang et al. [6].

Total genomic DNA was extracted from the needle tissues of each individual using Qiagen DNeasy plant mini kit according to the manufacturer's instructions (Qiagen, Toronto, ON, Canada). The quality and quantity of DNA was evaluated using a spectrophotometer and agarose gel electrophoresis.

### Isolation of Microsatellite DNA Sequences and Development of SSR Markers

An AFLP-based microsatellite-enriched library was constructed using total genomic DNA of black spruce accession 59 and applying the magnet beads method [29], [30], with minor modifications. Genomic DNA (∼600 ng) was digested with two different restriction endonucleases *Eco*RI and *Mse*I at 37°C for 2 h and ligated with *Eco*RI and *Mse*I adapters using the AFLP Analysis System I (Invitrogen Life Technologies, Burlington, ON, Canada). An initial PCR reaction was conducted in a total volume of 200 µl, containing 2.5 mM MgCl_2_, 0.16 mM each of dNTPs, 10 pmoles of *Eco*RI adaptor-specific primer (5′-GACTGCGTACCAATTC-3′) and 60 pmoles of *Mse*I adaptor-specific primer (5′-GATGAGTCCTGAGTAA-3′), 10 unit of Taq polymerase (Fermentas, Fisher Scientific, Ottawa, ON, Canada) and 10 µl of purified adaptor-ligated DNA as templates. Amplification was performed with 10 cycles each of denaturation at 90°C for 30 sec, annealing at 56°C for 60 sec and extension at 72°C for 60 sec. The amplification products were hybridized with 100 pmoles of 3′-biotin-labelled (AG)_15_ oligonucleotides, and the target DNA fragments were recovered using Dynabeads M-280 Streptavidin (Dynal Biotech, Life technologies, Burlington, ON, Canada) in a STEX buffer (100 mM NaCl, 10 mM Tris-HCl, 1 mM EDTA and 0.1% Triton-100, pH 8.0) for 30 min [29], [30]. We used (AG)_15_ probe for microsatellite enrichment because AG repeats were found to be most abundant in white spruce [2]. The enriched DNA fragments were amplified again with 25 cycles using the same cycling conditions. The purified PCR products were inserted into the pCR2.1 TOPO vectors and then transformed into One Shot Competent Cells provided in the TA cloning kit (Invitrogen Life Technologies, Burlington, ON, Canada), following the manufacturer's instructions. The bacteria were plated on LB agar plates containing X-gal and 100 µg/ml ampicillin. The positive clones (white and some light blue colonies) were screened by PCR using the Universal M13 Reverse Primer and the T7 Promoter Primers (Invitrogen Life Technologies, Burlington, ON, Canada). Colony blot hybridization was carried out using biotenylated (AG)_15_ probe and BrightStar BioDetect non-isotopic detection kit according to the user's manual (Ambion, Life Technologies, Burlington, ON, Canada) to further screen and identify the plasmids containing microsatellite sequences.

The putative microsatellite-containing clones were grown overnight in 3 ml of LB broth with 50 µg/ml ampicillin. Subsequently, plasmids were extracted using a QIAprep Spin Miniprep Kit (Qiagen, Toronto, ON, Canada). The purified DNA fragments were sequenced using the Universal M13 Reverse Primer labelled with IRdye 800 and the T7 Promoter Primer labeled with IRDye 700 with Thermo Sequenase fluorescent-labeled primer cycle sequencing kit (Amersham Pharmacia Biotech, Quebec, Canada).

The sequence data were analyzed using the E-SEQ v2 program and the Align IR program (LICOR, Lincoln, Nebraska, USA). Primers were designed for the non-redundant SSR-containing sequences using Primer 3 program [31]. The oligonucleotide primers complementary to the regions flanking the identified SSR repeat motifs were synthesized incorporating IRDye 700 or 800 labels by the Integrated DNA Technologies (Coralville, IA, USA), and used for amplification by PCR.

### PCR Amplification, Optimization and Visualization of Microsatellites

PCR amplification for all microsatellite markers was performed in a 20 µl reaction volume, with 100 ng of purified spruce genomic DNA, 1.5 mM of MgCl_2_, 200 µM of each of dNTPs, 200 ng of BSA, 1 U of *Taq* DNA polymerase (Fermentas, Fisher Scientific, ON, Canada) and 7.5 pmole of each forward and reverse primers. To optimize the amplification conditions for each microsatellite locus, the following temperature cycling parameters were tested: denaturation for 3 min at 94°C, followed by two cycles of 30 sec each at 94°C, 55–60°C and 72°C; and 38 cycles each of 15 sec at 94°C, 55–60°C and 72°C, with a final extension step of 10 min at 72°C. For the primers yielding no, weak or multi-locus amplification products using the above PCR profiles, a touchdown protocol was used: initial denaturation at 94°C for 3 min followed by two cycles each of 30 sec at 94°C, 60°C and 72°C; followed by 13 cycles each of 94°C for 15 sec, 60↓54°C for 15 sec (0.5°C per cycle) and 72°C for 15 sec; and 30 cycles each of 15 sec at 94°C, 54°C and 72°C, with a final cycle at 72°C for 10 min.

After amplification, the PCR products were separated on a 6.5% polyacrylamide gel at 1500 volts for 2.5 h on the LICOR sequencing system IR 4200 (LiCor, Lincoln, NE, USA) with IRDye 700 and 800 labelled size standards (IR 50–350 bp). The genotypes of spruce individuals were determined by their allelic constitution manually. The DNA fragment size of individual alleles was determined based on the size standard used.

### Informativeness, Inheritance and Linkage of Microsatellite Markers

The informativeness of the microsatellite loci was evaluated in black spruce and red spruce by determining the number of alleles and their frequency, observed heterozygosity(H_o_), and expected heterozygosity (H_e_). Also polymorphism information content (PIC) for each locus was calculated as follows [32]: 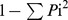 where *P*_i_ is the frequency of the *i*th allele in 30 individuals of a species examined. The PIC is the same as expected heterozygosity (H_e_).

The inheritance, segregation patterns and linkage of the polymorphic microsatellite markers were examined in two three-generation outbred pedigrees, which consisted of the parents and *F*_2_ progenies of two controlled crosses (32×40 (110 progeny), and 46×14 (30 progeny) of black spruce. The linkage relationships among the seven microsatellite loci (RPMSA04, RPMSA07, RPMSA09, RPMSA12, RPMSA13, RPMSA17, and RPMSA33), showing interparental polymorphisms in the 32×40 cross have been previously determined [6]. In the 46×14 cross, the two-point linkage was determined among five informative loci (RPMSA04, RPMSA11, RPMSA12, RPMSA26, and RPMS33), and between RPMSA13 and RPMSA22 by investigating joint segregation and independent assortment of their microsatellite DNA variants. Due to certain logistics reasons, three different sets of 30 *F*_2_ progeny of the 46×14 cross were used for determining the inheritance and linkage of the microsatellite markers: set 1 (RPMSA04, RPMSA11, RPMSA12, RPMSA26, and RPMS33), set 2 (RPMSA07), and set 3 (RPMSA13, and RPMSA22). Therefore, the pair-wise linkage relationships among all eight markers showing interparental polymorphism in this cross could not be tested. The null hypothesis that the two loci in a pair are independently assorted, was tested by the χ^2^ goodness-of-fit test. Since the expected number of progeny in genotypic classes for five microsatellite pairs (RPMSA04-RPMSA11, RPMSA11-RPMSA12, RPMSA11-RPMSA26, RPMSA11- RPMSA33, and RPMSA13-RPMSA22) was less than the minimum of 5 required for a valid χ^2^ test [33], we pooled adjacent two or four progeny genotypic classes to obtain a joint class with an expected progeny number >5 to perform the χ^2^ test [33]. This resulted in a total of two or four joint progeny classes.

### Cross-species Transferability of Microsatellite Markers

The number of alleles observed at each locus in eight individuals (five individuals for RPMSA07) of Norway spruce, Sitka spruce, white spruce, and Engelmann spruce were determined to test the cross-species transferability of the microsatellite markers developed from black spruce.

## Results

### Construction of Microsatellite-enriched Library and Identification of SSR-containing Sequences

The microsatellite-enriched library was constructed from genomic DNA of black spruce after restriction with a rare and a frequent endonucleases and enrichment with biotinylated (AG)_15_ oligonucleotides. One-thousand twenty-nine white and light blue colonies were isolated. Of these, 311 were identified as positive potentially containing DNA inserts after the first colony PCR screening with the T7 Promoter and the Universal M13 reverse primers. The second screening with the (AG)_15_ oligonucleotides as probes using non-isotopic detection method resulted in excluding 203 clones from the putative SSR-containing clones. Sequencing of the remaining 108 putative positive clones revealed 94 unique sequences containing microsatellite repeats with mono- (8.5%), di- (87.3%), tri- (3.2%), and tetranucleotide (1%) motifs (Table S1).

The DNA insert sizes of the 94 microsatellite-containing clones ranged from 148 bp to 1,273 bp, with an average of 462 bp. The majority of clones (77.4%) contained a moderate insert size of 200–600 bp, while five clones (BS-013, 019, 026, 236 and 245) contained larger DNA inserts (>800 bp) and three clones (BS-039, 119 and 241) smaller inserts (<200 bp). Thirty-nine (41.5%) of the identified microsatellites were perfect, 27 (28.7%) imperfect and 28 (29.8%) compound (Table S1). In terms of repeats, GA/CT motif was the most common, accounting for 84.6% of the perfect repeats. One clone (BS-236) contained a simple AT motif and the other two TG/CA motifs. A perfect trinucleotide (CTT) repeat motif was also found in one sequence (BS-120). Among the compound microsatellites, 25 were composed of two repeat types and three of three repeat types. Twenty-one out of 28 compound microsatellites were interrupted with one or more nucleotides (Table S1). The overall repeat motif number ranged from 4 to 54 units (Table S1).

### Microsatellite Markers and Their Informativeness in Black Spruce and Red Spruce

**Figure 1:**
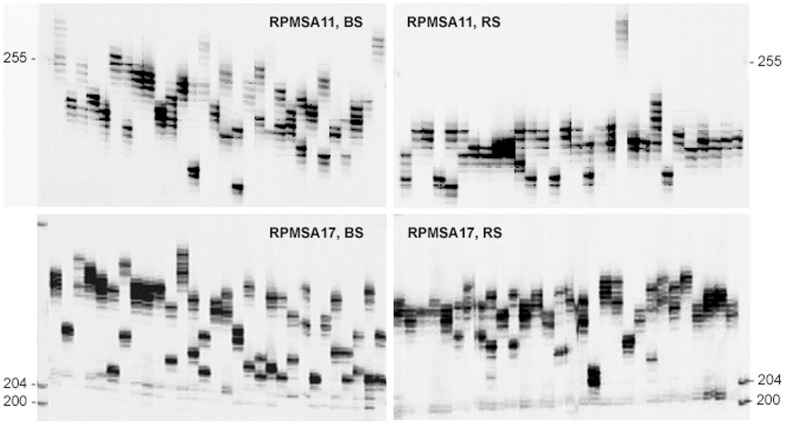
Allelic variation at two microsatellite loci. Allelic variation among black spruce (BS) and red spruce (RS) individuals at RPMSA11 and RPMSA17 microsatellite loci.

Primer pairs were designed from the flanking regions of microsatellites for selected 34 of the 94 well-characterized microsatellite-containing sequences (Table S2). Each of these primer pairs were initially tested for amplification of microsatellite DNA variants in three black spruce genotypes (59, 63 and N2). A variety of PCR conditions were tested. Of this set, 22 (64.70%) primer pairs yielded single-locus microsatellite variant patterns. The remaining 13 SSR primers either did not yield any amplicon or showed multiple DNA fragment patterns. Among the 22 microsatellite primer pairs resolving microsatellites with simple single-locus patterns, 16 primer pairs produced consistent and clear scorable amplicons in 30 black spruce individuals and 15 primer pairs in 30 red spruce individuals (Table 1; Table 2; Fig. 1). The optimal annealing temperatures for each of the 16 primer pairs are listed in Table 1.

**Table 1:** Polymorphic microsatellite markers/loci showing simple single-locus patters; and their repeat motifs, primer sequences, annealing temperatures, and GenBank accession numbers.

Microsatellite locus	Repeat type	Primers (5′-3′)	Annealing temperature (°C)	GenBank Accession No.
*RPMSA01*	(TA)_9_(GA)_7_(GATA)_13_	GGAGCTAAACACATTTGGTACAGG	60↓54	KJ847201
		GAAACCATTGATGTGGGTTG		
*RPMSA04*	(CT)_23_	GTGAGATTTTGGCAGCAACA	60	KJ847204
		TGATCACCCTTGCTCAAAGA		
*RPMSA05*	(GA)_20_	CCCTATTCCCACTTGAAATCC	60	KJ847205
		CTTATGGGCTCCACCACACT		
*RPMSA06*	(CT)_2_(GA)_6_	CTCGACAGACCCCTCTTTTG	60↓54	KJ847206
		CAAGTCTCGGTTCTCCTCCA		
*RPMSA07*	(CT)_16_	TGAAGAAGCAAGTGGGCTCT	60↓54	KJ847207
		TGCATTGATCTCTCCCCTTT		
*RPMSA09*	(AG)_8_	CACCTCAGTTCACACCTGCT	60	KJ847209
		TTCCTCTCCCAAGAATGTGC		
*RPMSA11*	(GA)_21_	GACCCTAGATTTTGGGGTAT	60	KJ847211
		CCCCCTCTCAGTAATCCAAC		
*RPMSA12*	(TC)_9_CC(TC)_4_	AACGAGGTTCATCCCATCTG	60↓54	KJ847212
		TACGCTCAATGTCGATGAGG		
*RPMSA13*	(GA)_9_	AACCATGAAACCCTAGCGACT	60	KJ847213
		TGAGGACTTAGGCCCACATT		
*RPMSA15*	(GA)_7_(AGAAT)(GA)_4_	ATCGATAGGCTTGCAAGAGG	60↓54	KJ847215
		TGTGCCCTCATGTGCTATGT		
*RPMSA17*	(CT)_12_	CAACGACTGCAACTGGGTACT	60	KJ847217
		CAACCATAGACACGCAACCA		
*RPMSA19*	(CT)_12_	TAGCCAATACAATGCCAAGG	60	KJ847219
		ATCAGAGCGAAGTTTGGAG		
*RPMSA22*	(TC)_6_/(TC)_14_/(GA)_4_	GCACGTGCATGTTCTCTGTC	58	KJ847222
		TGCATGCAGATGAATGAGAG		
*RPMSA26*	(CT)_2_C(CT)_9_	GCTGTAGGGTTGATATTTGC	65↓60	KJ847226
		TGTGTGAGAGATAAGTGTTGAG		
*RPMSA27*	(TC)_22_(TA)_19_	ATATTCGAATGAGAGAGCAATC	55	KJ847227
		TGTAGGCCCATGATAATGTA		
*RPMSA33*	(GA)_9_	ACACACATGAACACATGAGC	60↓55	KJ847233
		GCTGTATGGATTCCGTATGA		

**Table 2:** Informativeness of microsatellite markers in black spruce and red spruce.

Microsatellite locus		Black Spruce				Red Spruce		
	Number of alleles	Allele size range	H_o_	H_e_/PIC	Number of alleles	Allele size range	H_o_	H_e_/PIC
*RPMSA01*	1	206	0	0	1	170	0	0
*RPMSA04*	9	105–148	0.133	0.796	3	124–146	0.172	0.161
*RPMSA05*	17	165–231	0.267	0.909	Multilocus			
*RPMSA06*	1	208	0	0	3	206–210	0.172	0.587
*RPMSA07*	12	141–181	0.433	0.902	6	153–161	0.214	0.692
*RPMSA09*	4	164–170	0.400	0.576	6	146–200	0.217	0.617
*RPMSA11*	16	227–267	0.900	0.908	10	227–267	0.467	0.779
*RPMSA12*	3	185–202	0.100	0.661	5	184–206	0.267	0.639
*RPMSA13*	15	182–210	0.690	0.868	13	178–210	0.733	0.851
*RPMSA15*	7	192–203	0.900	0.745	4	197–209	0.900	0.663
*RPMSA17*	14	204–234	0.867	0.894	10	206–226	0.667	0.818
*RPMSA19*	8	137–151	0.467	0.57	4	139–145	0.167	0.541
*RPMSA22*	8	220–238	0.167	0.747	5	202–232	0.133	0.769
*RPMSA26*	2	119–121	0.067	0.34	3	119–123	0.200	0.636
*RPMSA27*	18	168–222	0.367	0.908	15	154–216	0.800	0.847
*RPMSA33*	15	176–216	0.667	0.868	5	192–208	0.600	0.662
**Mean**	**9.38**		**0.401**	**0.669**	**6.20**		**0.381**	**0.618**

H_o_, observed heterozygosity; H_e_, expected heterozygosity; PIC, polymorphic information contents.

In black spruce individuals, two loci, RPMSA01 and RPMSA06, both compound microsatellites (Table 1) were found to be monomorphic (Table 2). The numbers of alleles detected at the polymorphic markers were quite variable ranging from 2 (RPMSA26) to 18 (RPMSA27), with an average of 9.3 alleles per locus (Table 2). The DNA fragment sizes of alleles ranged from 105 bp to 267 bp (Table 2). Among the red spruce individuals, the RPMSA01 locus was monomorphic, while the RPMSA05 primers produced multilocus patterns. The number of alleles ranged from 3 to 15 at a locus in red spruce (Table 2), with a mean of 6.2 alleles per locus. The most informative locus in red spruce in terms of the number of alleles was RPMSA27, with 15 alleles detected in the 30 individuals. The frequencies of individual alleles at the polymorphic loci in 30 individuals of black spruce are provided in Table S3, whereas that for red spruce in Table S4. Most of the alleles had low frequencies as expected for highly polymorphic loci. In black spruce, the allele frequencies at a polymorphic locus ranged from 0.017 at 13 loci to 0.80 at RPMSA26 (Table S3). In red spruce, frequencies of individual alleles ranged from 0.017 at seven polymorphic loci to 0.588 at RPMSA09 (Table S4). Red spruce individuals had 30 alleles not detected in black spruce samples, whereas black spruce samples had 70 alleles not detected in red spruce samples at 15 loci (RPMSA05 was excluded because its primers produced multilocus patterns in red spruce and genotypes of individual samples could not be scored) (Table S3; Table S4). The expected heterozygosity or PIC values for the polymorphic microsatellite loci varied from 0.34 (RPMSA26) to 0.909 (RPMSA05), with an average of 0.67 in black spruce, and from 0.161 (RPMSA04) to 0.851 (RPMSA13), with an average of 0.62 in red spruce (Table 2). The observed heterozygosity values for the polymorphic microsatellite loci varied from 0.100 (RPMSA12) to 0.900 (RPMSA11 and RPMSA15), with an average of 0.401 in black spruce, and from 0.133 (RPMSA22) to 0.900 (RPMSA15), with an average of 0.381 in red spruce (Table 2).

### Inheritance and Linkage of SSRs in Black Spruce

**Figure 2:**
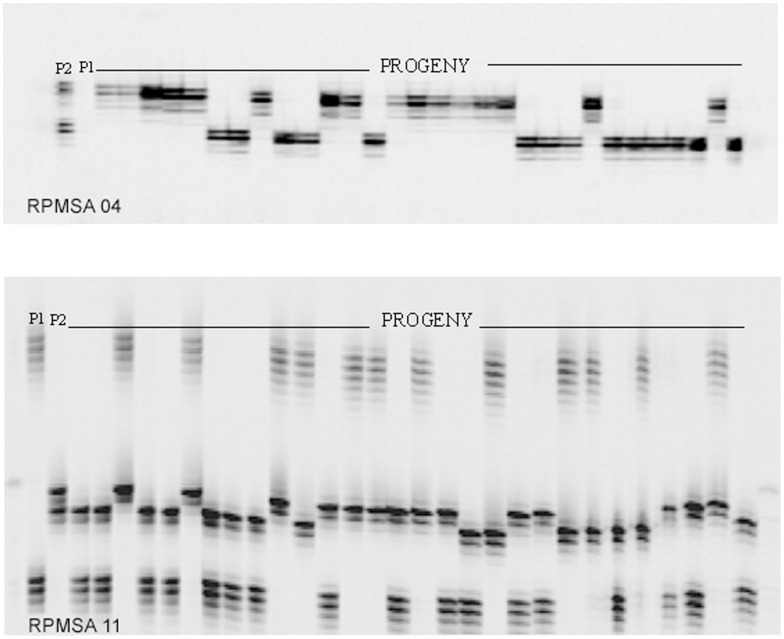
Inheritance of microsatellite DNA variants in black spruce. Inheritance and segregation pattern of microsatellite markers at (**a**) RPMSA04, and (**b**) RPMSA11 in *F*_2_ progeny of 46×14 controlled cross. The genotypes of the parents and progeny are provided in Table 3.

Ten microsatellite loci were found to be polymorphic between the parents of one or both controlled crosses (Table 3; Fig. 2). At each of the 10 loci, the progenies of each cross segregated into two to four genotypic classes as expected for single-locus Mendelian inheritance patterns (Table 3). However, significant deviations from the expected Mendelian progeny ratios were observed in two cases. Segregation of alleles at RPMSA04 in one of the two controlled crosses (46×14) conformed to the expected Mendelian ratios. However, the observed ratio in the progenies of the other cross (32×40) departed from the Mendelian expectations as evaluated by χ^2^-tests, *P*≤0.05 (Table 3). The second case was with RPMSA17 with four segregating alleles (Table 3).

**Table 3:** Inheritance of microsatellite markers in *F*_2_ progenies of two black spruce controlled crosses.

Primer/Locus	Cross	Parental genotypes	Number of progeny	Progeny genotypes (number)	Expected ratio	χ^2^	*P*
*RPMSA04*	32×40	*BO×AB*	110	*AO*(18)∶*BB/BO*(54)∶*AB(*38)	1∶2∶1	7.31	0.026
	46×14	*OO*×*AB*	30	*AO*(16)∶*BO*(14)	1∶1	0.13	0.715
*RPMSA07*	32×40	*AO*×*BC*	110	*AB*(33)∶*AC*(27)∶ *BO*(26)∶*CO*(24)	1∶1∶1∶1	1.64	0.651
	46×14	*AO*×*AB*	30	*AA(7)*∶*AO*(8)∶*(AB*(7)∶*BO*(8)	1∶1∶1∶1	0.53	0.912
*RPMSA09*	32×40	*AB*×*AA*	110	*AB*(61)∶*AA*(49)	1∶1	1.31	0.253
*RPMSA11*	46×14	*BC*×*AD*	29	*AB*(7)∶*BD*(7)∶*AC*(5)∶*CD*(10)	1∶1∶1∶1	1.76	0.624
*RPMSA12*	32×40	*AB*×*AA*	110	*AB*(49)∶*AA*(61)	1∶1	1.31	0.253
	46×14	*OO*×*AO*	30	*AO*(14)∶*OO*(16)	1∶1	0.13	0.715
*RPMSA13*	32×40	*AB*×*CD*	108	*AD*(31)∶*AC*(26)∶*BC*(20)∶*BD*(31)	1∶1∶1∶1	3.04	0.386
	46×14	*BC*×*AC*	30	*AB*(8)∶*AC*(9)∶*BC*(8)∶*CC*(5)	1∶1∶1∶1	1.20	0.753
*RPMSA17*	32×40	*AD*×*BC*	92	*AC* (27)∶*AB*(10)∶*BD* (36)∶*CD*(19)	1∶1∶1∶1	16.09	0.001
*RPMSA22*	46×14	*BB*×*AB*	30	*AB*(13)∶*BB*(17)	1∶1	0.03	0.853
*RPMSA26*	46×14	*BB*×*AB*	30	*AB*(14)∶*BB*(16)	1∶1	0.53	0.467
*RPMSA33*	32×40	*BC*×*AC*	104	*AC*(29)∶*AB*(30)∶ *BC*(17)∶*CC*(28)	1∶1∶1∶1	4.23	0.238
	46×14	*AB*×BB	30	*AB*(13)∶*BB*(17)	1∶1	0.53	0.467

Note: Only those microsatellite loci that showed interparental polymorphisms and segregation in the progeny are included. The alleles are coded alphabetically for simplicity to follow their inheritance and segregation. O = Null allele.

In the 32×40 pedigree, seven microsatellite loci showed independent assortment, and six of them were previously mapped on to six linkage groups (LG) [6]: LG01: RPMSA13; LG02: RPMSA09; LG04: RPMSA12; LG05: RPMSA33; LG06: RPMSA17; LG 07:RPMSA4. In the 46×14 cross, the Chi-square goodness-of-fit tests indicated that the observed numbers of genotypes were in agreement with those expected for independent assortment of two loci in each of the 11 two-locus combinations tested (Table S5).

### Cross-species Transferability of Black Spruce Microsatellite Markers in other *Picea* Species

Reproducible amplifications of the microsatellites of most of the 15 loci were obtained in Norway spruce, Sitka spruce, Engelmann spruce, and white spruce (Table 4). The fragments amplified from the four species were in the size range as observed in black spruce.

**Table 4:** Transferability of microsatellite markers to other four spruce species: number of alleles detected in selected individuals of each species.

Locus	Number of alleles			
	Norway spruce	Sitka spruce	Engelmann spruce	White spruce
*RPMSA01*	1	1	1	1
*RPMSA04*	2	8	3	1
*RPMSA05*	Multilocus	Multilocus	Multilocus	Multilocus
*RPMSA06*	1	1	1	1
*RPMSA07* *	6	5	4	5
*RPMSA09*	1	1	1	1
*RPMSA11*	3	10	10	9
*RPMSA12*	5	2	2	3
*RPMSA13*	6	9	9	7
*RPMSA17*	10	7	8	6
*RPMSA19*	1	1	1	1
*RPMSA22*	7	5	7	4
*RPMSA26*	-	-	-	-
*RPMSA27*	6	6	6	8
*RPMSA33*	-	1	5	4

Note:

*Five individuals each of *P. abies, P. sitchensis, P. glauca and P. engelmannii* were used to amplify the SSR locus RPMSA07, whereas eight individuals per species were used to amplify other 14 SSR loci.

- SSR locus not detected.

Among the 15 primers tested, nine yielded polymorphic amplicons in at least two species (Table 4). The RPMSA33 primers yielded polymorphic PCR products in Engelmann spruce and white spruce, a monomorphic product in Sitka spruce and no product in Norway spruce. The RPMSA05 primers detected several loci in Norway spruce, Sitka spruce, Engelmann spruce, white spruce and red spruce. The RPMSA01 SSR locus was monomorphic in all spruce species tested, including black spruce and red spruce. Interestingly, RPMSA06, RPMSA9, and RPMSA19 resolved polymorphic microsatellite DNA variants in black spruce and/or red spruce, but these loci were monomorphic in the individuals of other four species. While two or three alleles were detected at RPMSA26 in black spruce and red spruce samples (Table 2), the primers for this locus did not show any amplification in the four species.

## Discussion

### Isolation and Development of Microsatellite DNA Markers

We have developed highly informative single-locus genomic microsatellite DNA markers in black spruce and red spruce using an AFLP-based microsatellite enrichment and isolation method. This is the first report for microsatellite marker development in red spruce. A few genomic microsatellite markers have recently been reported for black spruce [34]. We demonstrate that 14 of the 16 microsatellite markers characterized are informative in black spruce and red spruce. Of these, seven (RPMSA05, RPMA07, RPMSA11, RPMSA13, RPMA17, RPMSA27, and RPMSA33) were the most informative loci in black spruce, each with over 10 alleles and expected heterozygosity/PIC close to 0.90. In red spruce, four loci (RPMA11, RPMSA13, RPMA17, and RPMSA27), with 10 or more alleles at a locus were the most informative. The levels of allelic polymorphisms of the microsatellite loci were positively correlated with the repeat numbers present in the original clones isolated from black spruce (r = 0.484, *p* = 0.04) and red spruce (r = 0.480, *p* = 0.04). These results are consistent with the positive correlation observed between the repeat length and the polymorphism in white spruce [2], Norway spruce [22], and other plants [35], [36]. In contrast, no such positive correlation between the microsatellite repeat length and resultant polymorphism was observed in some plant species [37], [38]. Microsatellite loci with compound repeats are generally believed to show lower polymorphism than loci with simple repeats. While this was true for two (RPMSA01, and RPMSA06) of the six microsatellite loci with compound repeats in our study, the locus RPMSA27 with compound CT and AT repeats showed the highest polymorphism in black spruce and red spruce (Table 2).

Several approaches have been used to isolate and develop microsatellite DNA markers from spruce species (Table 5). The comparison with other approaches (Table 5) suggests that the AFLP-based approach that we have reported here, provided the most efficient method for isolating and developing highly informative single-locus microsatellites with simple patterns in spruce. The success rate was over 250% higher than that was observed for microsatellite marker development in black spruce from standard microsatellite-enriched genomic libraries in our own lab (Table 5; Joy and Rajora, unpublished). It is worth noting that the success rate was even higher than that observed for isolating and developing microsatellites from cDNA and EST sequences [15] or from low-copy sequences selected from microsatellite-enriched genomic libraries [39] in spruce. We used *Eco*RI as one of the two restriction enzymes, which is methylation sensitive. The repetitive elements in the genome are highly methylated [40]. Therefore, methylated sites in the genome may have been preferentially excluded by using *Eco*RI and this may have enriched single- or low copy sequences in the microsatellite-enriched AFLP fragments library. Similar AFLP-based approach has also been successfully used to develop microsatellite markers in pear (*Pyrus pyrifolia*) [29] and peach (*Prunus persica*) [30].

**Table 5:** Comparison of development efficiency of single-locus or simple pattern microsatellite markers in *Picea*.

Species	Sources	Clones screened	Positive clones	Clones sequenced	Unique sequences	SSR containing sequences	Primer pairs designed (P)	SSR loci with simple patterns (S)	Rate of loci with simple pattern (%) (P/S)	Reference
*Picea mariana*	Enriched genomic DNA AFLP library	1029	108	108	103	94	34	22	64.70	This study
*Picea mariana*	Enriched genomic DNA library	2819	1612	120	90	73	40	10	25.00	Roy & Rajora (unpublished)
*Picea mariana*	Enriched genomic library		>200	20				7		Dobrzeniecka et al. [34]
*Picea glauca*	Non-enriched & enriched genomic library	5404	91	32		23	16	8	50.0	Rajora et al. [2]
*Picea glauca*	Enriched genomic library	200		60		56	34	13	38.23	Hodgetts et al. [21]
*Picea glauca & Picea sitchensis*	cDNA library EST sequences				34,846	188	44	25	56.81	Rungis et al. [15]
*Picea abies*	Enriched genomic library - trinucleotide repeats		121	100	100	85	55	23	41.82	Scotti et al. [22]
*Picea abies*	Enriched genomic library – dot blot selection of low copy number sequences	600	150	150		108	53	33	62.26	Scotti et al. [39]
*Picea abies*	Genomic library	∼65,000	223	46	46		36	7	19.44	Pfeiffer et al. [20]
*Picea abies*	cDNA library	50,000	23	23	23	16	16	6	37.50	Scotti et al. [61]

An AFLP-based method named Fast Isolation by AFLP of Sequences Containing Repeats (FIASCO) was recently introduced [10], which was believed to be a simple, rapid and effective method for microsatellite isolation from genomic DNA *de-novo*. However, this method did not prove effective in conifers as the percentage of microsatellite-containing clones was relatively low (between 29.5 to 43.1%) [23], [41], [42]. The FIASCO method does not apply a microsatellite enrichment step, whereas our method employs microsatellite enrichment followed by double screening for microsatellite-enriched clones. In our study, the enriched genomic library was screened with a colony PCR followed by a hybridization with (AG)_15_ oligonucleotides as probes. This most likely helped us to exclude the majority of non-microsatellite containing colonies and increased the efficiency of isolating microsatellite-containing clones. Of the 108 clones sequenced, 97 clones contained microsatellites and only nine (8.3%) did not. The sequences of two clones were of poor quality.

High allelic polymorphism and H_e_/PIC and simple single-locus inheritance patterns of the microsatellites developed in this study suggest that the developed markers could be used for various genetics, genomics, breeding, DNA fingerprinting, tree forensic and genetic resource conservation studies and applications in black spruce and red spruce. Indeed six (RPMSA04, RPMSA09, RPMSA12, RPMSA13, RPMSA17, and RPMSA33) of the 14 polymorphic microsatellite markers developed have already been mapped to six linkage groups in black spruce [6]. Thus, our study provides a rich resource of highly informative single-copy microsatellite DNA markers in spruce.

### Microsatellite Diversity and Divergence in Black Spruce and Red Spruce

Black spruce individuals had 150 alleles at 16 microsatellite loci and 133 alleles at 15 loci after excluding RPMSA05 that showed multilocus patterns in red spruce (Table 2). Red spruce individuals had 93 alleles at the same 15 SSR loci. Also, the expected and observed heterozygosity values were lower in red spruce individuals. This is consistent with previous reports based on allozyme and cDNA sequence-tagged sites (STS) markers that red spruce harbours relatively lower genetic diversity [43]–[45] as compared to sympatric black spruce and white spruce. Genetic diversity at the studied microsatellite loci could be regarded as high in black spruce and moderate in red spruce accessions studied. However, although for a limited number of samples, the microsatellite allelic variability results suggest that red spruce cannot be considered as genetically depauperate as reported by Perron et al. [44].

Red spruce individuals had a number of microsatellite alleles (30) that were not detected in black spruce individuals (Table 2; Table S3; Table S4). Also locus RPMSA06 was monomorphic for one allele in black spruce but had three alleles in red spruce individuals. Based on the STS markers, Perron et al. (2000) concluded that genetic diversity of red spruce is a subset of black spruce genetic diversity [44]. Our microsatellite results, albeit for a small but equal number of samples for black and red spruce, do not support this conclusion. The microsatellite locus RPMSA01 was monomorphic with species-specific alleles in black spruce (allele 206) and red spruce (allele 170). If these results hold true for a larger number of black spruce and red spruce individuals, the RPMSA01 locus could be used to unambiguously differentiate between closely related black and red spruce. These two spruce species hybridize naturally where their ranges overlap, and are difficult to differentiate morphologically. Also, no species-specific allozyme, ribosomal, mitochondrial or chloroplast DNA markers could be found for these species [46]–[48]. However, RAPD markers specific to black and red spruce have been reported [49]. But RAPDs are not reliable markers until converted to codominant and reliable SCAR markers. Therefore, codominant markers, such as the microsatellite DNA markers, could provide excellent tools to differentiate between black spruce and red spruce.

### Inheritance, Segregation and Linkage of Microsatellite Markers

The Mendelian inheritance and segregation of microsatellite variants in the progenies of two controlled crosses (Table 3) demonstrate that the variants at each of the 10 microsatellite loci are controlled by a single nuclear locus and that the microsatellite variants (alleles) at these loci are codominantly inherited in black spruce. Segregation distortions were observed at two microsatellite loci: RPMSA04, and RPMSA17 (Table 3). However, for RPMSA04, segregation distortion was observed in progeny of one controlled cross and not in that of the other. Segregation distortion or transmission ratio distortion of molecular markers has been reported in many plant species, including spruce [6], [50], [51]. In a black spruce-red spruce *BC*_1_ backcross, 47% of SAMPL (selectively amplified microsatellite polymorphic loci) markers showed distorted segregation ratios [50]. In Norway spruce, three (2.3%) out of the 133 tests with 51 SSR markers showed a significant deviation from Mendelian segregation ratios [51]. In other species, for example *Isis*, approximately 1/3 of all microsatellite markers in each linkage map revealed significant transmission ratio distortion [52]. In our study, two of 15 (13.3%) tests significantly departed from the Mendelian ratios. This level was comparable and falls within the range of distortion values observed in intraspecific crosses [53].

Segregation/transmission distortion is a common phenomenon in plants [54], with backcrosses and *F*_2_ showing high percentage of loci showing transmission ratio distortion [54]–[56]. Several biological mechanisms and experimental errors in scoring the mapping population genotypes can cause deviations from expected segregation ratios. Although we thoroughly verified marker scoring, one of the two parents in one of the two cases showing segregation distortions had null alleles. This may have caused some ambiguity in scoring the progeny genotypes. The presence of null alleles can cause segregation distortion in the crosses [57]. Biological mechanisms that can cause segregation distortion include chromosome/chromatin loss [54], divergence of the parental genotypes [53], [54], inbreeding depression, presence of the transmission distorter loci or genomic regions [55], meiotic drive locus [56], and genetic load and recessive lethal alleles [58]. We examined the inheritance of microsatellite markers in *F*_2_ progeny of black spruce. The parents of this progeny were related by descent. Back spruce shows high inbreeding depression [59]. Therefore, the observed segregation distortion may have been caused by inbreeding depression.

Our study in combination with the results reported by Kang et al. [6] suggests that there is no linkage among RPMSA04, RPMSA07, RPMSA09, RPMSA11, RPMSA12, RPMSA13, RPMSA17, RPMSA26, and RPMSA33, and between RPMSA13 and RPMSA22. Therefore, these microsatellite loci could be simultaneously used in population genetic studies in black spruce.

### Cross-species Transferability of Microsatellite Markers

Primers for 14 out of 16 microsatellite loci developed in black spruce were able to amplify microsatellites in at least four other species, with primers for 12 loci yielding amplification products in all six spruce species. In red spruce, primers for all 16 microsatellite loci worked, resolving single-locus variants for 15 loci and multilocus patterns for one microsatellite locus (Table 2). Thus, it could be inferred that most of the microsatellite markers developed from black spruce could potentially be used for various genetics, genomics and breeding studies in five other spruce species tested. The transferability rate in this study was higher than previously reported for spruce [2], [15]. It has been suggested that if a particular microsatellite locus could be resolved in black, white, red, and Sitka spruce by using the same primers, it is likely that the primers will be widely transferable to other spruce species as well [15]. The high cross-species transferability observed in the present study indicates that the regions flanking the microsatellite sequences are well conserved in the six spruce species studied. The cross species transferability of microsatellite markers in *Picea* species will reduce the cost of marker development.

The success of cross-species transferability of microsatellite loci depends on the evolutionary relatedness of the taxa being examined. In this study, primers developed for all 16 microsatellite loci from black spruce sequences resolved microsatellites in red spruce. This is consistent with close genetic and phylogenetic relationships between these two species [44], [60]. It is worth noting that primers for RPMSA05 resolved a single highly polymorphic microsatellite locus in black spruce but yielded multilocus patterns in red spruce, and other four spruce species tested.

## Conclusions

We have developed a reliable and highly efficient approach for isolating and developing highly informative microsatellite DNA markers with simple single-locus patterns in black spruce and red spruce. The markers inherited in a Mendelian single-locus codominant fashion and were not linked. Most of the microsatellite markers could be successfully transferred to Norway, Sitka, white, and Engelmann spruce. The new set of microsatellite markers developed here could be used for various genetics, genomics, breeding, DNA fingerprinting, tree forensics and genetic resource conservation studies and applications in six spruce and potentially other spruce species. The microsatellite markers reported here are based on developing primers for only 34 out of 94 unique SSR-containing sequences. Thus, additional markers could be developed from 60 microsatellite-containing sequences that we did not use.

## Supporting Information

Table S1 **Microsatellite-containing clones, repeat motifs and insert size in black spruce.**
(DOCX)

Table S2 **Microsatellite markers screened; their repeat motifs and primer sequences.**
(DOCX)

Table S3 **Allele frequencies at characterized microsatellite loci in 30 individuals of black spruce (** ***Picea mariana*** **).**
(DOCX)

Table S4 **Allele frequencies at characterized microsatellite loci in 30 individuals of red spruce (** ***Picea rubens*** **).**
(DOCX)

Table S5 **Joint two-locus segregation patterns and chi-square analyses for testing of linkage between microsatellite DNA loci in the 46×14 cross of black spruce (** ***Picea mariana*** **).**
(DOCX)

Table S6 **Microsatellite-containing sequences of black spruce used for primer design.**
(DOCX)
