# Supplementary material for: Highly Informative Single-Copy Nuclear Microsatellite DNA Markers Developed Using an AFLP-SSR Approach in Black Spruce (Picea mariana) and Red Spruce (P. rubens)
Source: PLoS One. 2014 Aug 15;9(8):e103789. doi: 10.1371/journal.pone.0103789 (PMC4134192; doi:10.1371/journal.pone.0103789)
Supplement: Table S1 — Microsatellite-containing clones, repeat motifs and insert size in black spruce. (DOCX) [file pone.0103789.s001.docx]

**Table S1.** Microsatellite-containing clones, repeat motifs and insert size in black spruce.

| **Clone #** | **Repeat motif** | **Primers developed and locus ID** | **Insert size** |
| --- | --- | --- | --- |
| BS-002 | (CT)_4_ |  | 584 |
| BS-006 | (GA)_4_(AG)_3_ |  | 280 |
| BS-009 | (GA)_4_ |  | 312 |
| BS-013 | (CT)_4_(TCT)(TC)_3_(TT)(TC)_3_ |  | 832 |
| BS-016 | (TA)_9_(GA)_7_(GATA)_13_ | *RPMSA01* | 335 |
| BS-018 | (GA)_5_ |  | 442 |
| BS-019 | (CA)_10_TA(CA)_10_(TA)_9_(GA)_19_AA(CA)_6_ | *RPMSA02* | 941 |
| BS-024 | (GA)_4_ |  | 501 |
| BS-026 | (CT)(CTT)_2_(CT)_4_ |  | 911 |
| BS-031 | (TG)_3_(TT)(TG) |  | 420 |
| BS-033 | (CT)_2_C(CT)_9_ | *RPMSA26* | 271 |
| BS-036 | (GA)_9_ | *RPMSA18* | 275 |
| BS-039 | (CT)_12_ | *RPMSA19* | 177 |
| BS-042 | (TC)_4_(GA)(TC)_2_ |  | 319 |
| BS-048 | (GA)_22_ | *RPMSA03* | 441 |
| BS-049 | (AT)_4_(ACCGACC)(AT)_5_ |  | 661 |
| BS-052 | (CT)_9_ | *RPMSA20* | 233 |
| BS-055 | (GA)_2_A(GA)_5_A(AG)_2_ |  | 293 |
| BS-058 | (GA)_13_ | *RPMSA21* | 333 |
| BS-061 | (AG)_5_ |  | 570 |
| BS-064 | (TC)_22_(TA)_19_ | *RPMSA27* | 306 |
| BS-067 | (AG)_4_ |  | 594 |
| BS-070 | (CT)_27_ |  | >600 |
| BS-072 | (AG)_4_(GA)_2_(AG) |  | 419 |
| BS-074 | (AG)_2_G(AG)_3_ |  | 529 |
| BS-077 | (GA)_3_(GGA)_2_(AG)_3_ |  | 310 |
| BS-078 | (CT)_23_ | *RPMSA04* | 442 |
| BS-079 | (GA)_3_(TC)(TT)(TC)_3_ |  | 491 |
| BS-082 | (GA)_20_ | *RPMSA05* | 471 |
| BS-084 | (CT)_2_(GA)_6_ | *RPMSA06* | 287 |
| BS-087 | (AG)_5_ |  | 527 |
| BS-090 | (CA)_6_ |  |  |
| BS-093 | (CT)_16_ | *RPMSA07* | 371 |
| BS-099 | (TC)_6_···(TC)_14_···(GA)_4_ | *RPMSA22* | 460 |
| BS-102 | (CT)_12_ | *RPMSA23* | 585 |
| BS-105 | (CT)_21_(CGC)(TA)_10_ | *RPMSA08* | 323 |
| BS-108 | (CT)_24_ | *RPMSA31* | 266 |
| BS-110 | (GA)_5_(AG)_2_(GA) |  | 438 |
| BS-113 | (CT)_5_ |  | 474 |
| BS-116 | (AG)_8_ | *RPMSA09* | 414 |
| BS-119 | (AG)_2_G(AG)_2_(CAT)(AG)_2_A(AG)_2_ |  | 189 |
| BS-120 | (CTT)_3_ |  | 306 |
| BS-122 | (A)_2_G(A)_4_G(A)_5_ |  | 492 |
| BS-123 | (CT)_17_(CA)_13_ | *RPMSA10* | 555 |
| BS-124 | (CT)_2_(TC)_5_(CT)(TC)_2_ |  | 223 |
| BS-127 | (GA)_4_(GC)(GA)_3_ |  | 287 |
| BS-130 | (CT)_2_A(CT)_5_(TC)_3_ |  | 420 |
| BS-131 | (GA)_5_ |  | 268 |
| BS-135 | (GA)_3_G(GA) |  | 556 |
| BS-139 | (AG)_6_(GT)(G)_3_(AG)_4_ | *RPMSA24* | 307 |
| BS-142 | (T)_13_ |  | 450 |
| BS-145 | (GA)_21_ | *RPMSA11* | 334 |
| BS-146 | (TC)_9_CC(TC)_4_ | *RPMSA12* | 431 |
| BS-151 | (GA)_9_ | *RPMSA13* | 358 |
| BS-155 | (GA)_4_(TA)(GA)_3_ |  | 547 |
| BS-158 | (GA)_6_ | *RPMSA32* | 618 |
| BS-161 | (CT)_5_C_4_(CT)_17_ | *RPMSA14* | 239 |
| BS-164 | (TC)_2_(CT)_3_(TC)_2_ |  | 641 |
| BS-167 | (A)_4_G(A)_4_ |  | 492 |
| BS-170 | (GA)_7_(AGAAT)(GA)_4_ | *RPMSA15* | 358 |
| BS-172 | (GA)_9_ | *RPMSA33* | 437 |
| BS-175 | (CT)_2_A(CT)_5_(TC)_3_ |  | 421 |
| BS-177 | (CT)_23_ | *RPMSA25* | 334 |
| BS-183 | (TC)T(TC)_3_T(TC)_2_ |  | 451 |
| BS-186 | (T)_6_(GT)_5_ |  | 507 |
| BS-192 | (CT)_19_ | *RPMSA16* | 406 |
| BS-195 | (TC)_4_ |  | 500 |
| BS-200 | (GA)_2_A(GA)_2_ |  | 648 |
| BS-201 | (TC)_4_ |  | 435 |
| BS-204 | (GA)_2_(AG)(GA)_5_ |  | 341 |
| BS-220 | (AG)G(GA)_4_ |  | 638 |
| BS-226* | (AC)_13_(AA)(AC)_12_(AGAGG)(GA)_22_ | *RPMSA28* | 278 |
| BS-230 | (A)_7_(G)_4_ |  | 686 |
| BS-233* | (AC)_13_(AA)(AC)_12_(AGAGG)(GA)_22_ | *RPMSA34* | 279 |
| BS-236 | (AT)_5_ |  | 942 |
| BS-239 | (TCAC)(TCAT)(TCAC)_3_(TGA)(TCAC) |  | 713 |
| BS-241 | (TTG)_2_G(TTG)_2_ |  | 148 |
| BS-242 | (AG)_5_ |  | 814 |
| BS-245 | (AG)_4_ |  | 1273 |
| BS-247 | (GA)_3_T(GA)_3_AA(GA)_2_ |  | 456 |
| BS-253 | (GA)_3_(AG)_4_ |  | 225 |
| BS-256 | (TC)_4_(CT)(TC)_2_ |  | 529 |
| BS-259 | (TG)_3_T(TG)_2_T(TG) |  | 339 |
| BS-262 | (A)_9_G(T)_5_ |  | 609 |
| BS-267 | (TG)_4_ |  | 518 |
| BS-269 | (GA)_17_ | *RPMSA29* | 409 |
| BS-276 | (T)_16_ |  | 769 |
| BS-277 | (AGG)_3_···(T)_5_G(T)_4_···(ACC)_3_ |  | 470 |
| BS-278 | (CT)_4_C(CT) |  | 432 |
| BS-281 | (TC)_6_(TT)(TC)_27_ |  | >360 |
| BS-287 | (CT)_12_ | *RPMSA17* | 410 |
| BS-301 | (A)_5_T(A)_6_ |  | 638 |
| BS-307 | (TA)_4_(AT)_4_(TA)_5_(AT)_2_A(AT)_2_A(AT)_28_ | *RPMSA30* | 691 |
| BS-310 | (GA)_2_(AG)_3_(A)_3_(GA)_3_ |  | 356 |
| BS-311 | (A)_7_C(A)_2_ |  | 416 |

*duplicate clones
