# Supplementary material for: Highly Informative Single-Copy Nuclear Microsatellite DNA Markers Developed Using an AFLP-SSR Approach in Black Spruce (Picea mariana) and Red Spruce (P. rubens)
Source: PLoS One. 2014 Aug 15;9(8):e103789. doi: 10.1371/journal.pone.0103789 (PMC4134192; doi:10.1371/journal.pone.0103789)
Supplement: Table S2 — Microsatellite markers screened; their repeat motifs and primer sequences. (DOCX) [file pone.0103789.s002.docx]

**Table S2.** Microsatellite markers screened; their repeat motifs and primer sequences.

| **Clone #** | **Locus/ Primer ID** | **Repeat motif** | **Primers (5’-3’)** |
| --- | --- | --- | --- |
| BS-016 | *RPMSA01* | (TA)_9_(GA)_7_(GATA)_13_ | GGAGCTAAACACATTTGGTACAGG |
|  |  |  | GAAACCATTGATGTGGGTTG |
| BS-019 | *RPMSA02* | (CA)_10_TA(CA)_10_(TA)_9_(GA)_19_AA(CA)_6_ | GCACCAGATAGTCCTACATAACCA |
|  |  |  | TCCCTCCCATGTGGAATTTA |
| BS-048 | *RPMSA03* | (GA)_22_ | CCAAGATGCTCCAAGAAACC |
|  |  |  | AGGAGAGTGTGCATTTCCTCTC |
| BS-078 | *RPMSA04* | (CT)_23_ | GTGAGATTTTGGCAGCAACA |
|  |  |  | TGATCACCCTTGCTCAAAGA |
| BS-082 | *RPMSA05* | (GA)_20_ | CCCTATTCCCACTTGAAATCC |
|  |  |  | CTTATGGGCTCCACCACACT |
| BS-084 | *RPMSA06* | (CT)_2_(GA)_6_ | CTCGACAGACCCCTCTTTTG |
|  |  |  | CAAGTCTCGGTTCTCCTCCA |
| BS-093 | *RPMSA07* | (CT)_16_ | TGAAGAAGCAAGTGGGCTCT |
|  |  |  | TGCATTGATCTCTCCCCTTT |
| BS-105 | *RPMSA08* | (CT)_21_(CGC)(TA)_10_ | TCATCACGTAGGCGTATCCA |
|  |  |  | CATGCATGCACACTAAATTG |
| BS-116 | *RPMSA09* | (GA)_8_ | CACCTCAGTTCACACCTGCT |
|  |  |  | TTCCTCTCCCAAGAATGTGC |
| BS-123 | *RPMSA10* | (CT)_17_(CA)_13_ | CATGGGGGAACATCCTTCTA |
|  |  |  | TGTAGGCTAGAACCCTTCATTG |
| BS-145 | *RPMSA11* | (GA)_21_ | GACCCTAGATTTTGGGGTAT |
|  |  |  | CCCCCTCTCAGTAATCCAAC |
| BS-146 | *RPMSA12* | (TC)_9_CC(TC)_4_ | AACGAGGTTCATCCCATCTG |
|  |  |  | TACGCTCAATGTCGATGAGG |
| BS-151 | *RPMSA13* | (GA)_9_ | AACCATGAAACCCTAGCGACT |
|  |  |  | TGAGGACTTAGGCCCACATT |
| BS-161 | *RPMSA14* | (CT)_5_C_4_(CT)_17_ | GGAACACGCACATGCTCTTA |
|  |  |  | TGAGGGAGACAGAGGGAGAT |
| BS-170 | *RPMSA15* | (GA)_7_(AGAAT)(GA)_4_ | ATCGATAGGCTTGCAAGAGG |
|  |  |  | TGTGCCCTCATGTGCTATGT |
| BS-192 | *RPMSA16* | (CT)_19_ | GGAAACCCTTTAGGGGAAGA |
|  |  |  | TGCAGAGCTGCTATTGGAGA |
| BS-287 | *RPMSA17* | (CT)_12_ | CAACGACTGCAACTGGGTACT |
|  |  |  | CAACCATAGACACGCAACCA |
| BS-036 | *RPMSA18* | (GA)_9_ | TTGAGCGATTCTCTCTCATCTG |
|  |  |  | CATGGGTTCCTCTCCATAGC |
| BS-039 | *RPMSA19* | (CT)_12_ | TAGCCAATACAATGCCAAGG |
|  |  |  | ATCAGAGCGCAAGTTTGGAG |
| BS-052 | *RPMSA20* | (CT)_9_ | TTAGGTCCCTAGCAAGATGA |
|  |  |  | TCTCAAACCTTCAAGGGATA |
| BS-058 | *RPMSA21* | (GA)_13_ | GAAGGGTATTCTACAACATCAC |
|  |  |  | GCCATTCCAACTCTTTGTC |
| BS-099 | *RPMSA22* | (TC)_6_···(TC)_14_···(GA)_4_ | GCACGTGCATGTTCTCTGTC |
|  |  |  | TGCATGCAGATGAATGAGAG |
| BS-102 | *RPMSA23* | (CT)_12_ | AGACCTTTAGGGTTCTTGCT |
|  |  |  | CACAAACTGCTAGAATGGTG |
| BS-139 | *RPMSA24* | (AG)_6_(GT)(G)_3_(AG)_4_ | GGGGTTATATGCAAGAGGTA |
|  |  |  | CTCTCTCCTTGTTTTCAGTCTC |
| BS-177 | *RPMSA25* | (CT)_23_ | ATGTCAGACCAAATTGAACC |
|  |  |  | GTGTACACAGATAAACACATGC |
| BS-033 | *RPMSA26* | (CT)_2_C(CT)_9_ | GCTGTAGGGTTGATATTTGC |
|  |  |  | TGTGTGAGAGATAAGTGTTGAG |
| BS-064 | *RPMSA27* | (TC)_22_(TA)_19_ | ATATTCGAATGAGAGCAATC |
|  |  |  | TGTAGGCCCATGATAATGTA |
| BS-226 | *RPMSA28* | (AC)_13_(AA)(AC)_12_(AGAGG)(GA)_22_ | ATTCTTATATTTACACACAC |
|  |  |  | TTCATCTCCTGGCCAACTGC |
| BS-269 | *RPMSA29* | (GA)_17_ | GAGGGGGAATAAAGAGAGAA |
|  |  |  | ATGTCTTGGACTCTCACACC |
| BS-307 | *RPMSA30* | (TA)_4_(AT)_4_(TA)_5_(AT)_2_A(AT)_2_A(AT)_28_ | ATGGGACTAGTGTGTTAGGG |
|  |  |  | AGTGTAGTGGTTGTGCGATT |
| BS-108 | *RPMSA31* | (CT)_24_ | CTGTGAAGGAAGGAAATCAG |
|  |  |  | ACGATTGACATGGAGAGAGA |
| BS-158 | *RPMSA32* | (GA)_6_ | GCCTAAATATGTTGGTGAGC |
|  |  |  | TACAACACATACGTCCCTCA |
| BS-172 | *RPMSA33* | (GA)_9_ | ACACACATGAACACATGAGC |
|  |  |  | GCTGTATGGATTCCGTATGA |
| BS-233 | *RPMSA34* | (AC)_13_(AA)(AC)_12_(AGAGG)(GA)_22_ | TTCTTATATATACACACACA |
|  |  |  | TTCATCTCCTGGCCAACTGC |
