# Supplementary material for: Highly Informative Single-Copy Nuclear Microsatellite DNA Markers Developed Using an AFLP-SSR Approach in Black Spruce (Picea mariana) and Red Spruce (P. rubens)
Source: PLoS One. 2014 Aug 15;9(8):e103789. doi: 10.1371/journal.pone.0103789 (PMC4134192; doi:10.1371/journal.pone.0103789)
Supplement: Table S3 — Allele frequencies at characterized microsatellite loci in 30 individuals of black spruce ( Picea mariana ). (DOCX) [file pone.0103789.s003.docx]

**Table S3.** Allele frequencies at characterized microsatellite loci in 30 individuals of black spruce (*Picea mariana*).

| **Microsatellite locus** | **Alleles** | **Corresponding frequency** |
| --- | --- | --- |
|  |  |  |
| *RPMSA01* | 206 | 1 |
| *RPMSA04* | 148,132, 128, 126, 122, 120, 110, 108, 104 | 0.017, 0.067, 0.200, 0.317, 0.100, 0.033, 0.216, 0.017, 0.033 |
| *RPMSA05* | 231, 225, 213, 197, 195, 193, 189, 187, 185, 183, 181, 179, 177, 175, 173, 171, 165 | 0.017, 0.017, 0.033, 0.017, 0.067, 0.033, 0.033, 0.083, 0.05, 0.083, 0.017, 0.067, 0.117, 0.050, 0.050, 0.033, 0.033, 0.200 |
| *RPMSA06* | 208 | 1 |
| *RPMSA07* | 181, 173, 169, 167, 159, 157, 155, 153, 151, 149, 147, 141, null | 0.017, 0.050, 0.050, 0.033, 0.150, 0.150, 0.083, 0.067, 0.033, 0.100, 0.067, 0.100, 0.100 |
| *RPMSA09* | 170,168,166,164 | 0.017, 0.484, 0.033, 0.433, 0.033 |
| *RPMSA11* | 267, 265, 263, 257, 255, 253, 249, 247, 245, 243, 241, 239, 237, 235, 233, 227 | 0.017, 0.050, 0.017, 0.033, 0.050, 0.133, 0.100, 0.117, 0.050, 0.117, 0.133, 0.083, 0.033, 0.017, 0.017, 0.033 |
| *RPMSA12* | 202, 200, 184, null | 0.017, 0.417, 0.233, 0.333 |
| *RPMSA13* | 210, 208, 206, 204, 202, 200, 198, 196, 194, 192, 190, 188, 186, 184, 182 | 0.017, 0.017, 0.259, 0.137, 0.052, 0.087, 0.035, 0.035, 0.103, 0.137, 0.017, 0.017, 0.035, 0.035, 0.017 |
| *RPMSA15* | 203, 201, 199, 197, 196, 194, 192 | 0.017, 0.367, 0.083, 0.300, 0.033, 0.067, 0.133 |
| *RPMSA17* | 234, 230, 228, 226, 224, 222, 220, 218, 216, 214, 212, 208, 206, 204 | 0.017, 0.050, 0.067, 0.184, 0.150, 0.083, 0.033, 0.033, 0.017, 0.033, 0.083, 0.050, 0.067, 0.133 |
| *RPMSA19* | 151, 149, 147, 145, 143, 141, 139, 137, null | 0.017, 0.017, 0.017, 0.033, 0.084, 0.033, 0.133, 0.633, 0.033 |
| *RPMSA22* | 238, 234, 232, 230, 226, 224, 222, 220, | 0.067, 0.067, 0.432, 0.150, 0.033, 0.067, 0.017, 0.167 |
| *RPMSA26* | 121, 119, null | 0.100, 0.800, 0.100 |
| *RPMSA27* | 222, 220, 216, 208, 206, 204, 202, 198, 196, 192, 190, 188, 186, 184, 182, 178, 168 | 0.017, 0.050, 0.033, 0.017, 0.050, 0.033, 0.033, 0.067, 0.067, 0.017, 0.067, 0.133, 0.200, 0.033, 0.033, 0.083, 0.017, 0.050 |
| *RPMSA33* | 216, 214, 212, 210, 208, 204, 202, 200, 198, 196, 194, 192, 190, 186, 176 | 0.017, 0.067, 0.050, 0.033, 0.033, 0.067, 0.033, 0.050, 0.017, 0.050, 0.017, 0.050, 0.282, 0.067, 0.150, 0.017 |
