# Supplementary material for: Highly Informative Single-Copy Nuclear Microsatellite DNA Markers Developed Using an AFLP-SSR Approach in Black Spruce (Picea mariana) and Red Spruce (P. rubens)
Source: PLoS One. 2014 Aug 15;9(8):e103789. doi: 10.1371/journal.pone.0103789 (PMC4134192; doi:10.1371/journal.pone.0103789)
Supplement: Table S4 — Allele frequencies at characterized microsatellite loci in 30 individuals of red spruce ( Picea rubens ). (DOCX) [file pone.0103789.s004.docx]

**Table S4.** Allele frequencies at characterized microsatellite loci in 30 individuals of red spruce (*Picea rubens*).

| **Microsatellite locus** | **Alleles** | **Corresponding frequency** |
| --- | --- | --- |
|  |  |  |
| *RPMSA01* | 170 | 1 |
| *RPMSA04* | 146, 126, 124 | 0.052, 0.035, 0.913 |
| *RPMSA06* | 210, 208, 206 | 0.362, 0.517, 0.121 |
| *RPMSA07* | 161, 159, 157, 155, 153, null | 0.053, 0.125, 0.161, 0.054, 0.107, 0.500 |
| *RPMSA09* | 200, 198, 196, 180, 164, 146, null | 0.043, 0.043, 0.043, 0.152, 0.588, 0.043, 0.088 |
| *RPMSA11* | 267, 245, 243, 241, 239, 237, 235, 233, 229, 227 | 0.033, 0.017, 0.017, 0.217, 0.067, 0.367, 0.100, 0.017, 0.150, 0.017 |
| *RPMSA12* | 206, 204, 202, 200, 184, null | 0.017, 0.133, 0.050, 0.033, 0.533, 0.234 |
| *RPMSA13* | 210, 208, 206, 204, 202, 200, 198, 196, 194, 192, 182, 180, 178 | 0.033, 0.033, 0.068, 0.100, 0.033, 0.168, 0.050, 0.050, 0.068, 0.300, 0.050, 0.033, 0.017 |
| *RPMSA15* | 209, 207, 205, 197 | 0.050, 0.250, 0.233, 0.467 |
| *RPMSA17* | 226, 224, 222, 220, 218, 216, 214, 212, 210, 206 | 0.100, 0.133, 0.333, 0.150, 0.117, 0.033, 0.067, 0.017, 0.017, 0.033 |
| *RPMSA19* | 145, 143, 141, 139, null | 0.033, 0.133, 0.650, 0.084, 0.100 |
| *RPMSA22* | 232, 230, 224, 220, 202, null | 0.033, 0.200, 0.133, 0.367, 0.167, 0.100 |
| *RPMSA26* | 123, 121, 119, null | 0.017, 0.300, 0.483, 0.200 |
| *RPMSA27* | 216, 214, 212, 210, 206, 202, 198, 196, 192, 178, 172, 170, 168, 165, 154 | 0.050, 0.017, 0.050, 0.050, 0.017, 0.017, 0.017, 0.033, 0.117, 0.066, 0.033, 0.017, 0.033, 0.150, 0.017, 0.317 |
| *RPMSA33* | 208, 206, 198, 196, 192 | 0.233, 0.282, 0.017, 0.450, 0.017 |
