# Supplementary material for: Highly Informative Single-Copy Nuclear Microsatellite DNA Markers Developed Using an AFLP-SSR Approach in Black Spruce (Picea mariana) and Red Spruce (P. rubens)
Source: PLoS One. 2014 Aug 15;9(8):e103789. doi: 10.1371/journal.pone.0103789 (PMC4134192; doi:10.1371/journal.pone.0103789)
Supplement: Table S5 — Joint two-locus segregation patterns and chi-square analyses for testing of linkage between microsatellite DNA loci in the 46×14 cross of black spruce ( Picea mariana ). (DOCX) [file pone.0103789.s005.docx]

**Table S5**. Joint two-locus segregation patterns and chi-square analyses for testing of linkage between microsatellite DNA loci in the 46 ×14 cross of black spruce (*Picea mariana*).

| **Locus combination** | **Parental genotypes**  **Locus1/Locus2** | **No. of**  **progeny** | **Number of progeny genotypes observed in each expected genotypic class**  **Locus1/Locus2** | **Expected ratio of genotypic classes** | **χ^2^ (*P*)** | **χ^2^Pooled_(df)_**  ***(P)*** |
| --- | --- | --- | --- | --- | --- | --- |
| *RPMSA4*/*RPMSA11* | *OO/BC* X *AB/AD* | 30 | 3 (*AO*/*AB*): 4 (*AO*/*AC*): 4 (*AO*/*BD*): 5 (*AO*/*CD*):  4 (*BO*/*AB*): 6 (*BO*/*AC*): 1 (*BO*/*BD*): 3 (*BO*/*CD*) | 1:1:1:1:1:1:1:1 | 4.80 (0.684) | 0.13 _(1)_  ( 0.715) |
| *RPMSA4*/*RPMSA12* | *OO*/*OO* X *AB*/*AO* | 30 | 10 (*AO*/*AO*): 6 (*AO*/*OO*): 4 (*BO*/*AO*): 10 (*BO*/*OO*) | 1:1:1:1 | 3.60 (0.308) |  |
| *RPMSA4*/*RPMSA26* | *OO*/*BB* X *AB*/*AB* | 30 | 8 (*AO*/*AB*): 8 (*AO*/*BB*): 6 (*BO*/*AB*): 8 (*BO*/*BB*) | 1:1:1:1 | 1.30 (0.729) |  |
| *RPMSA4*/*RPMSA33* | *OO*/*BB* X *AB*/*AB* | 30 | 6 (*AO*/*AB*): 10 (*AO*/*BB*): 7 (*BO*/*AB*): 7 (*BO*/*BB*) | 1:1:1:1 | 1.20 (0.753) |  |
| *RPMSA11*/*RPMSA12* | *BC*/*OO* X *AD/AO* | 30 | 3 (*AB*/*AO*): 4 (*AB*/*OO*): 5 (*AC*/*AO*): 5 (*AC*/*OO*):  3 (*BD*/*AO*): 2 (*BD*/*OO*): 3 (*CD*/*AO*):5 (*CD*/*OO*) | 1:1:1:1:1:1:1:1 | 3.15 (0.871) | 1.73 _(3)_ (0.630) |
| *RPMSA11*/*RPMSA26* | *BC*/*BB* X *AD*/*AB* | 30 | 4 (*AB*/*AB*): 3 (*AB*/*BB*): 5 (*AC*/*AB*): 5 (*AC*/*BB*):  1 (*BD*/*AB*):4 (*BD*/*BB*): 4 (*CD*/*AB*): 4 (*CD*/*BB*) | 1:1:1:1:1:1:1:1 | 3.06 (0.880) | 1.73 _(3)_ (0.630) |
| *RPMSA11*/*RPMSA33* | *BC*/*BB* X *AD*/*AB* |  | 2 (*AB*/*AB*): 5 (*AB*/*BB*): 5 (*AC*/*AB*): 5 (*AC*/*BB*):  4 (*BD*/*AB*): 1 (*BD*/*BB*): 2 (*CD*/*AB*): 6 (*CD*/*BB*) | 1:1:1:1:1:1:1:1 | 5.64 (0.582) | 1.73 _(3)_ (0.630) |
| *RPMSA12*/*RPMSA26* | *OO*/*BB* X *AO*/*AB* | 30 | 5 (*AO*/*AB*): 9 (*AO*/*BB*): 9 (*OO*/*AB*): 7 (*OO*/*BB*) | 1:1:1:1 | 1.47 (0.689) |  |
| *RPMSA12*/*RPMSA33* | *OO*/*BB* X *AO*/*AB* | 30 | 8 (*AO*/*AB*): 6 (*AO*/*BB*): 5 (*OO*/*AB*): 11 (*OO*/*BB*) | 1:1:1:1 | 2.80 (0.424) |  |
| *RPMSA26*/*RPMSA33* | *BB*/*BB* X *AB*/*AB* | 30 | 5 (*AB*/*AB*): 9 (*AB*/*BB*): 8 (*BB*/*AB*): 8 (*BB*/*BB*) | 1:1:1:1 | 1.20 (0.753) |  |
| *RPMSA13*/*RPMSA22* | *BC*/*BB* X *AC*/*AB* | 30 | 2 (*AB*/*AB*): 6 (*AB*/*BB*): 5 (*AC*/*AB*): 4 (*AC*/*BB*):  5 (*BC*/*AB*): 3 (*BC*/*BB*): 2 (*CC*/*AB*): 3 (*CC*/*BB*) | 1:1:1:1:1:1:1:1 | 4.15 (0.246) | 0.73_(3)_ (0.630) |
