# Supplementary material for: Highly Informative Single-Copy Nuclear Microsatellite DNA Markers Developed Using an AFLP-SSR Approach in Black Spruce (Picea mariana) and Red Spruce (P. rubens)
Source: PLoS One. 2014 Aug 15;9(8):e103789. doi: 10.1371/journal.pone.0103789 (PMC4134192; doi:10.1371/journal.pone.0103789)
Supplement: Table S6 — Microsatellite-containing sequences of black spruce used for primer design. (DOCX) [file pone.0103789.s006.docx]

**Table S6.** Microsatellite-containing sequences of black spruce used for primer design**.**

| **Clone #** | **Primer ID** | **Sequences** |
| --- | --- | --- |
| BS-016 | RPMSA01 | TATCTTCTAGGAATAAGTCATCAAACAATATCTT**GGAGCTAAACACATTTGGTACAGG**TTTGTGTTGTTGTATTTATGATATATATTTTTGGTTCAAGTTCCTCTTGTTGTCTATAATATATATATATATATATAGAGAGAGAGAGAGAGATAGATAGATAGATAGATAGATAGATAGATAGATAGATAGATAGATAGATATTTTTTAGGTATGAGTGGAAATATAAAGTTT**CAACCCACATCAATGGTTTC**TTATTGTTCGAAGGTTCTCCTAACACAAAAACATCAGATTGGTCAAACCCT |
| BS-019 | RPMSA02 | CACATCCAATGACAACATGGTGCATCCAATATAGTCGTGAATTCTAGGAGTTTTTGTCAAGTAGACAAACAAGCAAGAAAACTTGGAATGTATATAATATAGGTTGTTGTGAATTATTATTTGTAGTAGCAATGAACTACCTTTTTTGTTTTTTTGAA**GCACCAGATAGTCCTACATAACCA**TTGTTAGCCTTGCTACAACACACACACACACACACACATACACACACACACACACACACATATATATATATATATATAGAGAGAGAGAGAGAGAGAGAGAGAGAGAGAGAGAGAGAAACACACACACACACGCGAAAAATAAATAAA**TAAATTCCACATGGAAGTGGA**TTACGTACAACAATTGTCATTATCCATTATACCCATGGCATTATAATCTTCGTAAGTCATGCAAAGTTGTCATCTTTGTCTAACAATCTTATCCTCTTTCATTTAGGAATGACCTTTCTGCCAATGATCATTCTCTTTGGTGGTCACCTATTATTCTTGTTGTAGGAAAACCTTTTGCTAAAAGTTCTTGGTTATTGGTTTTGTGCTTTTTCCATAGAGTAATTATAACCCTTCAAGATATCTTCTTGTTTGAGAGTAAAGAATGGCTTTGCATGGACCTTTTCCAATAGAAAATTAGCTCATTATATTAGTGAAAGTATAATTTTTTACATCTTCATGACCCCACAAGAGTCCATTAGCAATACTACTCCATCATCATGCTCAAAATAATCATACTACGCAACACATCCCAGTACGCAAGTTGCAAACAAGTGATACAAAAAACAAGGCATAAAATCCCAACAGAAACCAGAAAAAGAAGCTAAACCAGTAGTCGCATGCCCTCAAAGGACCTCGAGTGTTTGCATGGACCTAG |
| BS-048 | RPMSA03 | GAAAGAGTAAGAAAGAGGAAATGCACACTCTCTTCTCTTAGTTACATCTCATCACAAGAGTGAATACATACGAGTGAATCAATATGTCGTAGAAAGACTCTCTCCTTCCGATTTTCTCTCAAAACTTATATCTCCAATTTCCAAGGGATAAACCCTAACCCTAGAACT**CCAAGATGCTCCAAGAAACC**GTACCACCTCGAGAGAGAGAGAGAGAGAGAGAGAGAGAGAGAGAGAGAGAGAGAGGTACAAGTGAAGACTTCAGAGAAAGA**GAGAGGAAATGCACACTCTCCT**CTCTTAGTCACTTCTCATCACAAGAGTGAATCAAAGTGAGTGAATCGATACGTTGTAGAAAGACTCTCTCTTTTCGTTTTTCTCTCAAAATCATAATCTTCAATTTCCAAGGGAAACC |
| BS-078 | RPMSA04 | ACAAAGTACCAACAAGAAGGAAGGTTCGGAAACAAACACAAAACTTATTTATTATAGGCTTGCCAAAGGGTTGATTGCACCACAATCAACAAGTCCAAAACCGGTACAA**TGATCACCCTTGCTCAAAGA**CAGAGTAGCTAGACTAGAGACGACTGAGATAAGAGAAAGAGAGAGAGAGAGAGAGAGAGAGAGAGAGAGAGAGAGAGAGAGAGAAAATATGTGT**TGTTGCTGCCAAAATCTCAC**CGCAATAATGTATCAAAACCTCCAATATATAGCCAAGAGAGAAGCACATCCCAATAAGAAACCAAGGAGGAAGCATAGGAGGTGGTAAGAGGAGTGTATGCGCATGTAGGATGAATAGGAATAGGGAAATACATGTGCACAAGGTAAGTTTGAGTGAGGAAGGTGAG |
| BS-082 | RPMSA05 | TTTCCTTATTGTAATATT**CCCTATTCCCACTTGAAATCC**ATGGTTTGATTAGTCCACATGTGTTACAGGAGAGAGAGAGAGAGAGAGAGAGAGAGAGAGAGAGAGAGAGTTTGATCAAGGGACCCTCCCAAGG**AGTGTGGTGGAGCCCATAAG**CACCAATCCACACTCCCCAGGTGTTGTTGAAGGCCCTTTTCATTGGTATAAGAATTATGTTGCTTCCCATTTTGTTGTTATTCCATTTGGAAAATTAGTTTGTAGTTATGTGTGTTTATTTTCCTTCTAGGTTGTAATAGCATTTGATTTCCTAACCCCACTAAACAATTGATTGTTCTCTGGCTTACATCTTGGCTATAGATTGTGATTTTTTCTCACTCGTGTTGATAAATCCAAAAAACATATTGGGGAATATAATATCCTATAGGTCCTAAATTCTTTCTCA |

| BS-084 | RPMSA06 | TTGAAGTTAGGTGGAGAGGACGTG**CTCGACAGACCCCTCTTTTG**TAAGGAAATCTTGCTTCAAATTTAGGAAGGTAGGATAGAGCGGAAGTAGGATAGAGAGTCTGTGTGGGACTCGAAGAAAAAAAAAAAGAGAGATAGGAGAGGTCTGGGTAGAGAGGCCTTAGGAAAGAGAAGCTCTGAGAGAGAGAGAGG**TGGAGGAGAACCGAGACTTG**TTATAGCCTAGTTATTGTAAACATCTGTATAGACATTCAGT |
| --- | --- | --- |
| BS-093 | RPMSA07 | GGCACCTCATGCTTGCTTCATACTTCAGTCACTGTTATCTAATTTTTTTTAGCATTGTTATGTTGCTTGGATGATTCTTGCATAATCATATTTCCCAAGTTATCATTGTTACACCCATAACTCAAAGCCTG**TGCATTGATCTCTCCCCTTT**GGAGAGTTTTTTACACACTTCTTGGTTCAAAGAGAGAGAGAGAGAGAGAGAGAGAGAGAGAGAAAAGGCCCCAAGGAACAACAAAGGACCCTCCCAAGGAAGAGAGG**AGAGCCCACTTGCTTCTT**CAATCCTCTACGGTGTATTCACATTTGTCTTACCATTGCTATACATTTGATCTTTGCTTGCAT |
| BS-105 | RPMSA08 | TGTAGAAGAATGAATGGCTTACAACAATCCTCTCAG**TCATCACGTAGGCGTATCCA**TGCACTGCACACATGCCTCTATGAGTATGCATAGATATATAACATCTCTCTCTCTCTCTCTCTCTCTCTCTCTCTCTCTCTCTCTCTCGCTATATATATATATATATATAGCATATATCAATAAATTATACTAACTGATTACAAACTCATCCATGAGTTACATAAACTGAT**CAATTTAGTGTGCATGCATG**CATATTCTATTTTTAGCTGATAGCTAACTATAGAATACTGAGAG |
| BS-116 | RPMSA09 | GGGGAGTGTTGAAATATTGTAACTACAAAGAGTATTCTATTTATTATTTGATGTGTTATTTCAATCCCCTTTTGTGGATTGATCATGTCGGGTATTGTAAATGACCAAATCTCCTTTTGTGGATTGGTCAAACCCTAATTATAATCACCCAAAATGAGAAGTCGTCCATGCTAGTGAAAAGA**CACCTCAGTTCACACCTGCT**AGATTGTATAAATAAGAGAGGCCAGCGCCTCATTTGTACACACACTCACTTGTTCATCAGAGCACAAGTTTGGCGTTGTAGTTGGAGAAGAAGGAAGAGAGAGAGAGAGAG**GCACATTCTTGGGAGAGGAA**TTTATGTATTGTTTGGTTTATTCTCTTGGCATTGTATTGGTTATCTATA |
| BS-123 | RPMSA10 | TGTTATTTTTTT**TGTAGGCTAGAACCCTTCATTG**TACTCTTTCACTATAATTGTACCCTCTCTCTCTCTCTCTCTCTCTCTCTCTCTCTCTCACACACACACACACACACACACACACTCACACCCACCAAGGAGAGCTAGTCTTAGGATTATCTCATCTCATCTTGTCTTCTGAATTGGATATATTG**TAGAAGGATGTTCCCCCATG**GAATTGGATTGTATTCATTGACTTTTTATCATTTTCAAGGCACCCAGTTCATAACATTTTATTTCACAACATTGATTCTAGGGTTTTTATTCTTCAAACGAGTCATCTTGAAATTTTCACTATAACTTTTTTGGGGGATGCTTCAGACAACAAAAAAATTTGGTTACTTAGGAGACATATCCAAGTCCTTCTAGTAAACAATCCAGGTTCTTGTTTGTTAT |
| BS-145 | RPMSA11 | CTAAAGATGGCTATGCATGCATTGTAATGGATTATTTTGGGAAAGATAGTAATAGAACTTGAG**ACCCTAGATTTTGGGGTAT**AAATACGTAGAGAGAGAGAGAGAGAGAGAGAGAGAGAGAGAGAGAGAGAGATGTATGCGTAAACAAGTGCGAGAGGGTGCGCATGAAAGAGAATTGAGGTTATGGAAAGATCTTGAATGTAATAGTAAGTTAGTAAGAAAGAAGATGAATAAAGAAAAAGTTTAGAAAGAATATTGTT**GTTGGATTACTGAGAGGGGG**GGTGAATCAGTAATAGAGCAAT |
| BS-146 | RPMSA12 | CAAATACATCATGTATC**AACGAGGTTCATCCCATCTG**TGTCGATACATTACATATTGGAAAAGTTCCTTCCTTCCTTTCTGATACATAGTTTATTAGAAAGGTTTCTCTCTCTCTCTCTCTCCCTCTCTCTCCCATGCATTACTTATCCATGATTTTCCCTCTTCTATCGTTGATGTGCCAGTAGAAAGATGTTGACC**CCTCATCGACATTGAGCGTA**TCGATAATGATCTTCTTTTCCTCACTGACATAGACCTTGTCGGCAAGACATGCCTTCATCTTGAGAAATCTCCTATTCTCTTCCAGCTCAACTCCTTCTCGATGTGCACATCGGTCAAGTTCCTATCATTTCCAATGATTTCATGTTTGACTAGATAACTGAGGACTGATGCCTCCCTCAA |

| BS-151 | RPMSA13 | GGGATAAACCCTAACCCTAGATTTCAAAATGCTCTAAGATGCTCAAAGA**AACCATGAAACCCTAGCGACT**CTGAGAGAGAGAGAGAGAGTACAAATGAACACCAAGAGAGAGAGAGAGAGAGAGGGAAAAGACTAGTGGGCCTAAGTCCTTCTCTTATGAATGAGAAGGACTCTCTCCTTCCTCTATATATATGAGAGGTAAGGGAGGCCAACAATAC**AATGTGGGCCTAAGTCCTCA**CAAGGAACATACAATAAGCTCATGTAGGCCTACATAAGTGAAACACTACACTCCAAGGGTACACATGTCATGCTTCAAGGTGATGTCG |
| --- | --- | --- |
| BS-161 | RPMSA14 | GATCACGCGCACACTCTA**GGAACACGCACATGCTCTTA**TAAGTCATTCCCTCATCTCTCTCTCCCCCTCTCTCTCTCTCTCTCTCTCTCTCTCTCTCTCTCCCCTAAATCTACCCACCCTTTCT**ATCTCCCTCTC**ACTCTCCCTCTATACCCCCCTGTCTATCTCCCTCTGTCTCCCTCACACTTCTATACATGGTAATACTTATG |
| BS-170 | RPMSA15 | AAGAATGACAAATCAGATAAACAAAATACCGACAAGAAACAAGGTTAGAACACAAACACTTCATTTCTTT**ATCGATAGGCTTGCAAGAGG**GTTGGGTGCACCACAATTGGCAAGTCCACAATCGGTACAATGATCACTTTGCTCAAAGAAGAGTCACTAGACTAAGAGAGAGAGAGAGAAGAATGAGAGAGAAACACTATGCTGCTGCTGCCAAGAACCGACCAAGAACCAATGGAATCCATCCATT**ACATAGCACATGAGGGCACA**TATCCAAGGAAAAAGTATGAAGACACATGGAGTAAACAAAAGAAAGGGTGAGTTCTCAG |
| BS-192 | RPMSA16 | TCCATTATTATAGTTCTTTTTATTTCAATGGATAACAACAACATAAGACAAGATCACTGAGAAATACACAATCACAAGAGACACATCATTTATCTTG**GGAAACCCTTTAGGGGAAGA**AAGCCCAGAAAAACTTCCTTATTATAGATAGCTAATACAATGCCAAGAGATAACTCAAGCTAATACAAAAGATCCTCTCCCAAATATGCACCTCTCTCTCTCTCTCTCTCTCTCTCTCTCTCTCTCTCT**TCTCCAATAGCAGCTCTGCA**ACTTGTCTCTCAATGAGAAAGTGTGGTGTGTCTAAGTGAGGTGCTAGCCTCCTTATTTATACTAATCTGGCACGTGTGAAGTGTCTCTTCACTGGCAGGGCCGACTTC |
| BS-287 | RPMSA17 | TATTGAAGTATTATATGCAGAAAAGAACAGGTCAACCCTACTTGAAGAGACACATGTTCACTTGGCCATATTAGTATAAATAGAAGGTGCTTGAGATCGTTTGGTGACACACTCACACTCAAGAAAGTGATGTGAAATTCTAGAGGAAGAGTGCAGCTG**CAACGACTGCAACTGGGTACT**GGTCTTGGTCTGTGGTTTGGGACTTTATGCTTGGTGAGATACCTTTGGAGAAAGGTGCTCTCTCTCTCTCTCTCTCTCTCTTGGCTATCATTTTGGTCTAGTGTTTCTTCTATAATCGTTCTATCTGTCAAAGGTTACAATGATTGTGGG**TGGTTGCGTGTCTATGGTTG**GGTGACAATATTGTGATATTGTTTATGG |
| BS-036 | RPMSA18 | AGGGAAGCGTACTTGCATATTGTAAACAGATC**TTGAGCGATTCTCTCTCATCTG**TAGAAGGTAGAGGCATTTATGTGTAGTAAATGAAAGTAGGTAGGTTTGAATGAAGATTAGACAATTTAGGAGCCTAGGAGGGAAAACCAACTATAGACACCCTCACCTTGGTATCATTGTAACATTAGAGAGAGAGAGAGAGAGATAACAGAGA**GCTATGGAGAGGAACCCATG**TATAGAAGTTTTGAA |
| BS-039 | RPMSA19 | TATAGA**TAGCCAATACAATGCCAAGG**GAATAAACCAAACAATACAGAAAGTACTCTCCCAAGTAGTGCATCTCTCTCTCTCTCTCTCTCTCTCTTCTTGTCCAACTGCGG**CTCCAAACTTGCGCTCTGAT**GATAAAGTGTGTGAG |
| BS-052 | RPMSA20 | TGTTGGCCTTTATTATATAGAAAACATATTATTCCATCTACTTGAGGGGTTAGGGAGG**TTAGGTCCCTAGCAAGATGA**CCTAGAGAAGAGTGCACACTAGTCATCTCTCTCTCTCTCTCTCCTCAAGTTGCTAGGGTTTCTTAGTTTTCCTGGAGCATCTTGGAAGTCTAGGTTTAGGGTT**TATCCCTTGAAGGTTTGAGA** |

| BS-058 | RPMSA21 | CAA**GAAGGGTATTCTACAACATCAC**TAGGAGAGAGAGAGAGAGAGAGAGAGAGAGGGAAACAACATCAACAGTAACTGGAACCCTAACTGGCATATCTATAAATCATCGAAAGTTTTTCTCATCATCAATATCATCATTCAC**GACAAAGAGTTGGAATGGC**ATCATCATCATCTGCATCAACATGGATTAGTATAATATTAATGAGTTGGTGTGTTATTTTTTTAGGTCATAAATCCTATGCATCTAACCCATGTATGACCCATAAGTCATGACACTAAAACCATCAATTTTAGTAAATTA |
| --- | --- | --- |
| BS-099 | RPMSA22 | **TGCATGCAGATGAATGAGAG**AGAAAGATACACACACACACACACACAGACCATGGCTTTACTACTAGAGAGAGAGATACCATGACTTTACTACAGGTCTCTCTCGCACATTAGAGAGAGAGAGAGAGAGAGAGAGAGAGACCATGGCTTTACTACTAGAGAGAGAGAGATACCACGACTTTACTACAGGTCTCTCTCGCACATTAGAGA**GACAGAGAACATGCACGTGC**ACACGTGATCTCTCTATCTAATGTGAGAGAGACCTCTAATTAGTGCACATGCATGAGAGAGAGAGAGAGAGAGAGAGATACGATGACTTTACTACTAGAGACGGAAACTGTGACTTTACTATAGGTCTCTCTCTCACATTAGAGAAAGAGAGAATGTGTATGTTCGACAACACAAGTATTCCTACAGAGGATGAACACC |
| BS-102 | RPMSA23 | AGTTATTTATTTATGTAAAAATACCATCCTTTTGGCCTCTTGTATTGGCTTTGGTGAATGAGCGTAGCTGCCATCTCTCTCTCTTCATAGATAAGCTACCACCATTCTAGCTAGTTTTTGTTCTATAATATTTTGCTATTTTATTCACGAATAGAACTTGGGTTTTCATTGTATTATTATTCTCTCTACTATATTTATGCGTTTGGTTCATTTCGTTCCCTATACAATCCACTATTTTATCATAGTCCTCAGTAAGAGATTTCCAACGAACTACTATGCACGCGAAACATAGATTGCATGCGAAAGTTACGCACCTGGAAAATTGATGTAGAAACTAACGCCTAAAAATAACTTTTGGCACTTCTCCCCCCTAAGTTTAGGAATTT**AGACCTTTAGGGTTCTTGCT**TTATGTAAAAAGGAAATCATTTTATCCTTTTATATTGGCTTTGGTGAATGAGCGTAGACACCATCTCTCTCTCTCTCTCTCTCTCTCTTCAGAGATAAGCTAC**CACCATTCTAGCAGTTTGTG**TTCTAAATATCCGCTATTTTATTCA |
| BS-139 | RPMSA24 | AGAGAGCAAGGTGGCGAGAGTGGGGGAGAGAGAGGGATGAATATACAAGTAAGAGATWGCG**GGGGTTATATGCAAGAGGTA**AAGAGAGAGGGGGGTAGATATGGATTGTTTGAGAGTGTGACAGGGTGAGAGAGGAAGACCAAGATAGGGGTAGAGAGAGAGAGGTGGGAGAGAGAGTGAGACT**GAGACTGAAAACAAGGAGAGAG**ATAAAGAGGTGATAGATGTAGGAGAGAAAGAGGGTGGAGATGGTGGGAGGGAGGGACAAAGGGGAAGA |
| BS-177 | RPMSA25 | GCGTAAGTTTTTATATATACAAGTGTTTTTCCGTTATTCACAACTAAGTGTTCTTCTTTCGTTTCATATCTTTTACGAAAAATCATACAGTTACAGGAAAGAGCCTTTGTTTGTAGTCCTATCTCGTTCTTCACACAAAGTTTTCTCTC**ATGTCAGACCAAATTGAACC**AGCCATTAAATGTAAATTATTTTTTCTTGTTTTATGGCTTTCCCTCTCTCTCTCTCTCTCTCTCTCTCTCTCTCTCTCTCTCTCTCTCTCCACACACATATAT**GCATGTGTTTATCTGTGTACAC**ACACAATT |
| BS-033 | RPMSA26 | CCGCCAGTGTGATGGATATCTGCAGAATTCGGCTTAT**TGTGTGAGAGATAAGTGTTGAG**TATGAGAGAGAGAGGAGGGGAGAGAGAGAGAGAGAGAGGAGAGAAAGAATATATATTGGGT**GCAAATATCAACCCTACAGC**TGGGTGCGCAGGGAGGGGAGAGAGAGAGAGGAGAAGAAAGAAGATATACTAGCTGAGAGCGCAGACAAGAACTGTACTCTTCATC |
| BS-064 | RPMSA27 | CTCCAAAATAGTACTCATACATCATTTTAGATATGTATCATTATGTATCTCACTTATTT**ATATTCGAATGAGAGCAATC**ATCTCTCTCTCTCTCTCTCTCTCTCTCTCTCTCTCTCTCTCTCTCTATATATATATATATATATATATATATATATATATATACCTTCCTTATAGATGTATTGAGTAATAAACTTTATAGGTCAACCATAAATATAA**TACATTATCATGGGCCTACA**TAACTAAACTGACATGACATAAAGTGACCACTATAAA |
| BS-226 | RPMSA28 | ATATAAATCT**ATTCTTATATTTACACACAC**ACACACACACACACACACAAACACACACACACACACACACACACAGAGGGAGAGAGAGAGAGAGAGAGAGAGAGAGAGAGAGAGAGAGAGAGAGTTAGATCTAAGTACAAGAATTATTCAACATTTACTGTCACTAAAAACAAATTATAGGTAAAGATATTAGTTTGTGCCTGA**GCAGTTGGCCAGGAGATGAA**TCGACCATCTTTCACTCCAGTG |

| BS-269 | RPMSA29 | TTTCCTTTGCTTGGCATGCCAAGCCTTGCCATGTCATGCCTTGCACATTCCTATGAGAATCTATGAGAGAGGCATAGTGAGAGGAAGGAAAGCAGAGAGGGAGACATAGAAGGGGGGCTAAAGAGAGAGTGAGAGGAAGGGGGGATACAGAGGGAGATAGA**GAGGGGGAATAAAGAGAGAA**AGGGGGGTATAGAGAGTGAGATTGAGACTAACAATGAGGAGAGAGAGAAAGGGTGGGAGAGAGGTGGAAGAGAGAGGGGAGGGAGGGACAAAGTGATAGAGAGAGAGAGAGAGAGAGAGAGAGAGAGAGAGATAATTGAAGAGGGGGGGATAGGGACGGAGTG**GGTGTGAGAGTCCAAGACAT**GTCAAGGCAAGGT |
| --- | --- | --- |
| BS-307 | RPMSA30 | CATGCACACTCTAGAGATAGGAGAAATAATTCAAACCATTTCCTCCAGCCGTTTTCCCTCCTCCGGTGCAAACCTGTGTGCCCTACTCTTCTGCAAATATGTGTGACCTCCTCCTCTGCAAATCCGTGTGTCCTCCTCTACAAATCCATCTTCCCTAGCCTCCTCTCGCATGCCCTCCTCCCGTGTGCCTCTCCCTAATTCTCTTCCTTCTCCCCCTTCCACTCTGTGTGCTAATGATCGTGCCCTAGCCTCCTCCCATGCGCCTATCCCTCCTTCTCCTCCTTCTCTCGTCCTCCTCTCCCTTTTGTGCTCCAAATCTATGCTCCTATGTATAGAGGTGTGCGACCATGGTGGTGAGTGAGAGAAGCTCTGCGTGTGATCCAAATCCGAGCTCCTATGTATAGCGGTGTGCGACTTTGGTGGTGAGTGTGAGCAGCTCTATGCACG**AGTGTAGTGGTTGTGCGATT**TGAGTTTCATTTTGTTTTGTTTGTATGTTGTTTTATTTTCATTTTTATTTTTCATTTTTATTATTATTTATATATAATATATATTATATATATAATATAATATAATATATATATATATATATATATATATATATATATATATATATATATATATATATATAAT**CCCTAACACACTAGTCCCAT**ATAAATGCA |
| BS-108 | RPMSA31 | TCCGAAGCTTCATCCAAGGAAGGACAAAAGGATTGTGCATGTACTCAGGAAGGAAGAAAGATTACCAATGAAGAAAGAAGAACA**CTGTGAAGGAAGGAAATCAG**CCACTTGGTTTTGCCAGAAGTGGATGTCACAACTTCATTGTATATTGCTTCCATTAAATGAGCATGCTCTCTCTCTCTCTCTCTCTCTCTCTCTCTCTCTCTCTCTCTCTCTCTCCATGTCAATCGTAGT |
| BS-158 | RPMSA32 | CACAGTGACTGATACTGGGTCGATTGAGCAGAAGAGAAAAAAATACGGTGTGAAGTAAGAGAAAGAAAGTTTTGGACGAGGATATCGAGGATGGATAGATCATCAAGAAGGATCATGGAAAACTCAGCAAGCCGAAGCCATTGTTGGATGCTTTGGAGCACCGCACGAGAAGAAAGGAAAGATAGGCTGGTGTAGCCCTGAAAACGTGGGTCAAGTTGACCTAGGGTCTCAGACACTTAGGGTTTTGGTATCTAGGTTTTTGACACTTAGGTTTTG**GCCTAAATATGTTGGTGAGC**TATTGTAATGTATAAATAAGAACCTAGATGAGGGAAGAGGATTGGTTCCAAATTGTCATTGCTATGTGTGATTATTGTTGGGAGAGAGAGAGAAGCATTGAGCAG**TGAGGGACGTATGTGTTGTA**AGATCTTCAGTTGGGTGCGAAGATCAACCCTGCAGTTGGGTGCATAGGGAGGGAGGACTGAGAGATGATAAAAGAGAGAAAGAGAGAGGATCTACTAGCCAAGAGTGCATATAAGAACTGTACGCTTTCATC |
| BS-172 | RPMSA33 | ACTTGGCGTAATCTCAAAAAAAGATTAGTGTTTTGGTTCCAGCTAAAACTATAAAGAAAAGTCAAGATAGAGAGTCCAA**ACACACATGAACACATGAGC**AGTTGAGCAGGATGAGCCACTGAGAGAGAGAGAGAGAGAGCCAGCCAAGAAAGAGGTGTTTCCAGGAAACACACAATTCCATACAAGTATGGAAGTCCATACAGTTGTACAAATAGAACTGTATGGATCTGTATTG**TCATACGGAATCCATACAGC**AATGCATCTATTGCAAATGACTTGGGGTGATGGCCTCATAGTGAGGGGGCGTTGCTCATGCTTTGTATCCTTTGTTTTTTTTTTACATTTCTTTCTTTTCATTTTGCTTTGCATCTTCATCTTTGCATCTTGCTTTTTCCCTTTTGTTTA |
| BS-233 | RPMSA34 | ATATAAATCTA**TTCTTATATTTACACACACA**CACACACACACACACACAAACACACACACACACACACACACACAGAGGGAGAGAGAGAGAGAGAGAGAGAGAGAGAGAGAGAGAGAGAGAGAGTTAGATCTAAGTACAAGAATTATTCAACATTTACTGTCACTAAAAACAAATTATAGGTAAAGATATTAGTTTGTGCCTGA**GCAGTTGGCCAGGAGATGAA**TCGACCATCTTTCACTCCAGTG |

**Note 1) Underline: primer sequences 2) Highlighted: microsatellites**
